# Combining machine learning and physiological network models for sepsis prediction

**DOI:** 10.3389/fnetp.2026.1852577

**Published:** 2026-06-19

**Authors:** Juri Backes, Artyom Tsanda, Tobias Knopp, Wolfgang Renz, Eckehard Schöll

**Affiliations:** 1 Institute for Biomedical Imaging, Hamburg University of Technology, Hamburg, Germany; 2 Section for Biomedical Imaging, University Medical Center Hamburg-Eppendorf, Hamburg, Germany; 3 Business Channel Diagnostics, Fraunhofer Research Institution for Individualized and Cell-based Medical Engineering IMTE, Lübeck, Germany; 4 Faculty of Electrical, Media and Information Engineering, Hamburg University of Applied Sciences (HAW Hamburg), Hamburg, Germany; 5 Institut für Physik und Astronomie, Technische Universität Berlin, Berlin, Germany; 6 Bernstein Center for Computational Neuroscience Berlin, Humboldt-Universität, Berlin, Germany

**Keywords:** coupled oscillator, dynamical systems, electronic health records, hybrid modeling, network physiology, sepsis onset prediction

## Abstract

As the most extreme course of an infectious disease, sepsis poses a serious health threat, with a high mortality rate and frequent long-term consequences for survivors. Despite its enormous burden on global healthcare and ongoing research efforts, early sepsis onset prediction remains challenging due to the complex nature of its pathophysiology. Current approaches face a fundamental trade-off: data-driven machine learning models achieve strong performance but lack interpretability, while biologically inspired models provide mechanistic insights but have limited clinical validation. In this study, we propose the *Latent Dynamics Model*, a hybrid machine learning approach that integrates a functional model of coupled oscillators representing organ- and immune-cell populations and their interactions. Here, the model parameters encode physiological conditions and allow for an interpretable differentiation between healthy and pathological states. By projecting high-dimensional patient data into the low-dimensional parameter space of the functional model, machine-learned trajectories through this space allow the prediction of critical organ system states and simultaneously offer interpretability beyond plain risk estimates. The proposed method is trained and evaluated on real intensive care patients, achieving competitive AUROC/AUPRC performance on a retrospective MIMIC-IV cohort. Additional qualitative analysis reveals that the learned trajectories exhibit clinically plausible patterns of deterioration, recovery, and stability. We demonstrate that a physiological network model can be embedded within a deep learning architecture without compromising predictive performance while providing an interpretable latent structure for sepsis onset prediction.

## 1 Introduction

The growing field of physics-informed machine learning allows to integrate Hermann Haken’s vision of synergetics—the study of how coherent macroscopic order emerges from interacting microscopic components (Haken, 1977; Haken, 1983) —into the age of data-driven prediction approaches. In the clinical context of sepsis, one of the most prominent manifestations of systemic self-organization and breakdown, this approach offers a powerful means to bridge mechanistic understanding and predictive modeling. In the systemic disorder of sepsis, where the coordination among physiological subsystems falters, we face a paradigmatic challenge of synergetic dynamics. Building on the framework of adaptively coupled phase oscillators that has been successfully applied to model dynamic transitions in physiological networks (Sawicki et al., 2022; Berner et al., 2022), we propose a physics-informed learning architecture that integrates this nonlinear network model within machine learning algorithms. This coupling of model-based and data-driven paradigms extends synergetics introduced by Haken (1988), translating his concepts of order parameter dynamics and self-organization into computational schemes capable of advancing real-time clinical prediction.

Nearly 20% of all deaths worldwide and approximately 11 million deaths annually stem from sepsis, a life-threatening organ dysfunction caused by a dysregulated host response to infection (Rudd et al., 2020). Despite overall advances in medical care and the slowly decreasing prevalence numbers, sepsis continues to be the leading cause of in-hospital death (La Via et al., 2024). Positive treatment outcomes are highly dependent on timely recognition and intervention (La Via et al., 2024). Each hour of delayed treatment increases the mortality risk, underscoring the critical importance of early detection (Seymour et al., 2017).

Traditional sepsis screening relies on clinical scoring systems such as sequential organ failure assessment (SOFA) or qSOFA. Although these scores are useful for standardizing assessment, they are inherently reactive, identifying patients already experiencing organ dysfunction rather than those at risk of it. This clinical reality has motivated the development of automated prediction systems, which continuously monitor patients and predict elevated sepsis risks based on predetermined clinical criteria derived from laboratory measurements. These automated alerts act as early warning systems, potentially allowing clinicians to initiate antibiotic or other treatments more rapidly or intensify patient monitoring.

With the increasing availability of electronic health records and computational resources, machine- and deep-learning methods have become the dominant paradigm for sepsis onset prediction systems. Despite enormous effort and reasonable predictive performance, interpretability and clinical adoption remain largely unsolved. Research on data-driven sepsis onset prediction systems is highly active; in the past 5 years alone (2021–2026), six systematic reviews on data-driven sepsis onset predictions have been published (Bomrah et al., 2024; Moor et al., 2021; Yadgarov et al., 2024; Gao et al., 2024; Parvin and Saleena, 2023; Stylianides et al., 2025). The reviews include a total of 180 studies (7–73 works per review), proposing more than 50 distinct methodologies, ranging from classical to highly specialized methods. Across studies, reported area under the receiver operating characteristic curve (AUROC) values typically range from 0.60 to 0.95, indicating modest to excellent performance, although such values must be interpreted cautiously given differences in cohort definition, task formulation, and evaluation protocols.

Clinical implementation studies consistently identify barriers, such as alert fatigue, where excessive false positives or clinically non-actionable alarms disrupt workflow, reduce clinicians’ trust, and ultimately lead to ignored warnings. These challenges reflect two fundamental limitations inherent to current data-driven approaches. First, prediction systems face a fundamental trade-off: higher sensitivity captures more true cases but generates more false alarms, while higher specificity reduces alert fatigue but risks missing sepsis cases where early treatment is critical. Second, the explainability of these methods predominantly relies on Shapley-value analyses, deriving importance factors of single input features or input-feature interactions (Stylianides et al., 2025; Sundararajan and Najmi, 2020). In addition to the generation of risk estimates, these *post-hoc* explainability analyses offer minimal additional insights for clinical practice, hindering adoption and acceptance among clinicians (Eini-Porat et al., 2022).

In contrast, mechanistic models explicitly encode biological processes, providing interpretability, but they require detailed parametrization and high-resolution measurements rarely available (Relouw et al., 2024; Cockrell et al., 2023), thereby prohibiting large-scale validation and clinical adoption. What is needed is an approach that inherits the scalability of data-driven methods while retaining the interpretability of mechanistic models.

**FIGURE 1:**
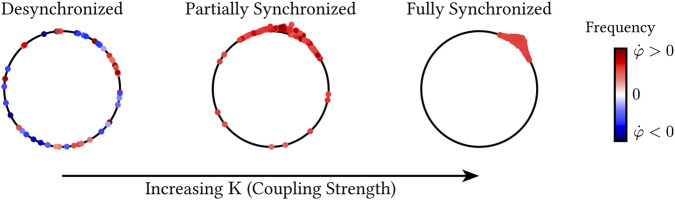
Illustration of the collective dynamics of a population of phase oscillators on the unit circle as the global coupling strength K is increased. Each point represents an oscillator at phase $\varphi_{i}$ ; color encodes the instantaneous phase velocity ${\dot{\varphi }}_{i}$ . For weak coupling (left), oscillators are desynchronized, with phases distributed around the circle and heterogeneous frequencies. At intermediate coupling (center), partial synchronization emerges: a subset of oscillators forms a coherent cluster that becomes phase- and frequency-locked, while the remaining oscillators drift incoherently. For sufficiently strong coupling (right), the population becomes fully synchronized, with all oscillators sharing a common phase and frequency (for clearer visualization the points are slightly dispersed).

Recent studies have introduced a physiological network model (Sawicki et al., 2022; Berner et al., 2022), with a few empirical parameters as a middle ground, a functional model that describes sepsis-related organ dysfunction via dynamically coupled oscillator networks, thereby representing organ- and immune-system interactions. The model is medically motivated but phenomenological rather than microscopic at the biochemical or cellular level, avoiding the need for high-resolution measurements. In the model, two key parameters, a biological age including comorbidities and the strength of organ–immune coupling, govern transitions between synchronized states and desynchronized regimes, where synchronization reflects coordinated organ function, while desynchronization corresponds to the breakdown of physiological regulation observed in sepsis. Synchronization and desynchronization are schematically depicted in Figure 1.

Although the model demonstrates rich theoretical behavior aligned with pathophysiological understanding, it has never been empirically validated on patient data.

To address this gap, we propose the Latent Dynamics Model, a hybrid, i.e., physics-informed, approach that combines the strengths of data-driven and model-based approaches for sepsis onset prediction. Rather than treating sepsis as a black-box classification problem, patient organ states are represented as trajectories through the parameter space of the physiologically inspired oscillator model, providing a proxy for acute organ failure. Unlike simple scoring systems, this trajectory-based approach can capture the direction of patient state change, not just its current value.

Conceptually, we map electronic health record time series to interpretable components aligned with the technical Sepsis-3 definition (Singer et al., 2016). The proposed model is trained retrospectively on real intensive care patient data, from the MIMIC-IV database (Johnson et al., 2023a; Johnson et al., 2023b). By precomputing dynamics over the parameter space, we enable gradient-based training without requiring computationally expensive integration of ordinary differential equations at each step. We assess the prediction performance quantitatively and evaluate interpretability qualitatively by analyzing generated patient trajectories.

Our contributions are as follows: (1) empirical demonstration that the physiological network model is compatible with real intensive care unit (ICU) data, (2) the Latent Dynamics Model architecture combining *gated recurrent unit* (GRU; Cho et al., 2014)-based electronic health records encoding with physiological network model parameter projection and predictions aligning with the Sepsis-3 definition, (3) an efficient differentiable lookup enabling end-to-end training, and (4) qualitative trajectory analysis demonstrating clinically plausible behavior, i.e., consistent representation of deteriorating, recovering, and healthy patients.

The remainder of this paper is structured as follows: Section 2 introduces the technical Sepsis-3 definition, the physiological network model and its pathology, followed by a description of its clinical interpretation. In Section 3 we present the new Latent Dynamics Model as an approach to the prediction problem, its architecture, and detailed description of its components. Section 4 describes the Latent Dynamics Model training, the data resource, preprocessing, and evaluation methodology. Quantitative results are discussed, and individual patient trajectories are analyzed, in Section 5. Current limitations and future research directions are discussed in Section 6.

## 2 Physiological network model of sepsis

In this section, the state of research is presented, with a focus on the definition of sepsis and a functional model based on network dynamics.

### 2.1 Definition of sepsis

Sepsis is defined as a life-threatening organ dysfunction caused by a dysregulated host response to infection (Singer et al., 2016) and cannot be reduced to a single specific physiological phenomenon. It is a multifaceted condition of physiological, pathological, and biochemical abnormalities, where patient progressions are largely heterogeneous (Singer et al., 2016). In addition, its triggers are explicitly nonspecific, suggesting that vastly different infections can result in the same physiological collapse. This makes it notoriously difficult to study the underlying pathophysiologic processes.

To provide an understanding of the processes driving a septic condition, we introduce two broad cell families that constitute the human organ tissue: the *parenchyma* and the *stroma*, which are separated by a thin, specialized boundary layer known as the *basal lamina*. Parenchymal cells provide the physiological functionality of an organ; in contrast, stroma cells are found in the structural and connective tissue, including blood vessels and nerves. The stroma not only contributes to tissues structure but actively participates in biochemical signaling and immune regulation, helping maintain a healthy and balanced state, the *homeostasis*. It further enables coordinated immune responses to injury or infection (Honan and Chen, 2021).

When a pathogen enters the body through the skin, a mucous membrane, or an open wound and is detected by specialized “detector cells,” the first line of nonspecific defense, the innate immune system, is activated, releasing signaling proteins called *cytokines* (Fischer and Deindl, 2022). Cytokines act as molecular messengers that coordinate the recruitment of circulating immune cells and guide them to the location of infection or injury (Zhang and An, 2007).

Under normal circumstances, the release of inflammatory cytokines is tightly regulated in time and magnitude. The release peaks as immune cells are recruited and automatically fades out once the initial pathogen is controlled and the host returns to homeostasis. In certain scenarios, a disturbance in the regulatory mechanisms triggers a positive inflammatory feedback loop, accompanied by a massive release of pro-inflammatory cytokines. These cytokines further activate additional immune and non-immune cells, which, in turn, amplify cytokine production, creating a continuous, uncontrolled, and self-reinforcing cycle of immune activation (Jarczak and Nierhaus, 2022).

With this overreaction, called *cytokine storm*, immune responses and release of inflammatory mediators may damage the body more than the infection itself. This condition is not restricted to infected areas but can also affect surrounding parts of the tissue and circulation systems, causing localized inflammatory response to become systemic. Widespread cytokine reaction starts to disrupt normal metabolism of parenchymal cells in organs due to a deficiency in oxygen and nutrients.

To compensate, cells switch from their usual oxygen-based metabolism to an *anaerobic* metabolism, generating energy less efficiently from glucose (Lamsfus-Prieto et al., 2016). As a result, metabolic by-products such as lactate accumulate, making the surrounding environment more acidic, further harming the cells and leading to more cellular dysfunction. At the same time, the mitochondria start to fail, and the walls of blood vessels become leaky, allowing fluids to move into surrounding tissue, thereby causing swelling and lowering blood pressure. These mechanisms further reduce the cellular oxygen supply (Jarczak et al., 2021).

Step by step, the deterioration of cells spreads throughout the body and affects organ functionality. When multiple organs fail simultaneously, the condition becomes increasingly difficult to reverse (Singer et al., 2016). A patient at this stage has progressed toward septic shock, the most severe form of sepsis, in which mortality increases drastically with each additional affected organ. Pin-pointing the moment at which the immune response switches from normal to dysregulated behavior remains difficult. To operationalize this complexity for clinical use, formal criteria have been developed. In this study, we follow the Sepsis-3 definition, the most up-to-date and widely used sepsis characterization. According to the Sepsis-3 definition, a patient is in a septic condition if the following two criteria are fulfilled: (i) confirmed or suspected infection and (ii) dysregulated host response (organ functionality). To measure the degree of dysregulation, the Sepsis-3 consensus relies on the SOFA score introduced by Vincent et al. (1996), Singer et al. (2016). The SOFA score is calculated at least every 24 h and evaluates six different organ systems by assigning a score from 0 (normal function) to 4 (high degree of dysfunction) to each. The overall score is calculated as the sum of each individual organ system. An increase in the SOFA score of ≥2 , in consecutive assessments, corresponds to an acute worsening of organ functionalities and a drastic worsening of the patient’s condition, which is used as an indicator of a dysregulated host response.

### 2.2 Model

In the spirit of *Network Physiology*, which considers the human body as a complex, integrated system, where emergent macroscopic dynamics arises from interactions among the diverse organ systems and subsystems (Ivanov, 2021), a functional physiological network model for sepsis has been proposed by Sawicki et al. (2022), Berner et al. (2022). It is a two-layer network model of simple phase oscillators with adaptive coupling, where one layer models the organs (parenchymal layer) and the second layer models the innate immune system, and the cytokines are modeled by adaptive interactions. This functional model is referred to as the physiological network model and forms the conceptual foundation for this work.

The Kuramoto phase oscillator model provides a paradigmatic minimal description of synchronization phenomena in complex systems (Kuramoto, 1984; Pikovsky et al., 2001). It consists of N∈N identical, fully connected, and coupled oscillators with phase $\varphi_{i}\in \left.\left[0,2\pi \right.\right)$ , for i∈1 …N and an intrinsic frequency $\omega_{i}\in R$ .

**FIGURE 2:**
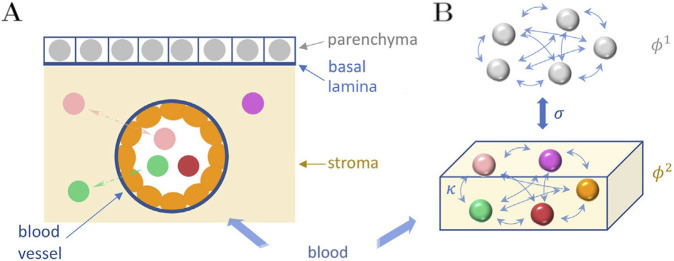
Schematic illustration of the two-layer physiological network model. **(A)** A tissue element, showing the parenchymal layer of organ cells (grey), and the immune layer (stroma, yellow), where a capillary blood vessel and various cells involved (colored, e.g., fibroblasts, macrophages, leukocytes, and thrombocytes) are depicted. The two layers are separated by the basal lamina. **(B)** Functional interactions within (κ) and between (σ) the two corresponding network layers are denoted by the phase variables $\varphi^{1}$ and $\varphi^{2}$ , respectively. Adapted from Berner et al. (2022).

A schematic illustration of organic tissue consisting of parenchymal cells and immune cells is shown in Figure 2. Panel A depicts the initial configuration of a tissue element. The tissue element consists of the epithelial parenchyma, the basal lamina, and the stroma. The parenchyma is the organ-specific functional layer. In the stroma blood supply, lymphatic drainage and immune response occur. The stroma consists of an extracellular matrix composed of collagen, glycoproteins, proteoglycans, and water, along with embedded cells (resident fibroblasts and fat cells, along with mobile cells such as macrophages, mast cells, granulocytes, and plasma cells). With the blood supply via capillaries, pro- and inflammation-inhibiting molecules are delivered to the stroma of each organ. They originate from the primary focus of infection, from the reticuloendothelial system, and from the innate immune system (macrophages and leukocytes). The pathophysiological positive response pattern is the maintenance of the anti-inflammation balance. Under a pathological condition, cytokines interact with the parenchyma and reduce parenchymal function via impairment of mitochondrial cellular respiration, eventually ending in organ failure.

Panel B shows the functional interactions in the two-layer network model of the organ tissue (parenchymal layer) and the stroma (immune layer). The network layer of parenchymal cells (superscript 1) are represented by N phase oscillators $\varphi_{i}^{1}$ , where i=1,…,N , and the network layer of immune cells (superscript 2) are represented by N phase oscillators $\varphi_{i}^{2}$ . The coupling weights in the parenchymal layer are considered to be partly fixed and partly adaptive, while in the immune layer, the coupling weights are completely adaptive. We model the communication through cytokines, which mediate the interaction between the parenchymal cells by the coupling weights $\kappa_{ij}^{1}$ and those between the immune cells by coupling weights $\kappa_{ij}^{2}$ . Note that $\varphi_{i}^{2}$ and $\kappa_{ij}^{2}$ represent the collective dynamics of all dynamical units of the stroma. Hence, this set of variables can be regarded as collective dynamical variables used in our functional modeling approach. The use of phase oscillators for the functional modeling of the interacting parenchymal cells and immune cells is motivated by the fact that phase oscillator networks are a paradigmatic model for collective coherent and incoherent dynamics. The healthy state is assumed to be characterized by regular periodic, fully synchronized dynamics of the phase oscillators. Healthy and pathological cells differ by their metabolic activity, i.e., pathological cells shut down their mitochondrial cellular respiration and switch to anaerobic glycolysis. Therefore, they are less energy-efficient and thus have a modified cellular metabolism and reduced function, which is reflected in our phase oscillator model by a different frequency, and the system splits into multifrequency clusters. Generally, multifrequency clusters often emerge as intermediate regimes between full synchronization and incoherence (Berner et al., 2019a).

We consider a general duplex network with two layers, each consisting of N identical adaptively coupled phase oscillators. Their dynamics is described by the following coupled ordinary differential equations (Equation 1):

$$\begin{array}{cc} {\dot{\varphi }}_{i}^{1} & =\omega^{1}-\frac{1}{N}\overset{N}{\underset{j=1}{\sum }}\left\{\left(a_{ij}^{1}+\kappa_{ij}^{1}\right)\sin\left(\varphi_{i}^{1}-\varphi_{j}^{1}+\alpha^{11}\right)\right\}-\sigma \sin\left(\varphi_{i}^{1}-\varphi_{i}^{2}+\alpha^{12}\right)\\ {\dot{\kappa }}_{ij}^{1} & =-\epsilon^{1}\left(\kappa_{ij}^{1}+\sin\left(\varphi_{i}^{1}-\varphi_{j}^{1}-\beta \right)\right)\\ {\dot{\varphi }}_{i}^{2} & =\omega^{2}-\frac{1}{N}\overset{N}{\underset{j=1}{\sum }}\left\{\kappa_{ij}^{2}\sin\left(\varphi_{i}^{2}-\varphi_{j}^{2}+\alpha^{22}\right)\right\}-\sigma \sin\left(\varphi_{i}^{2}-\varphi_{i}^{1}+\alpha^{21}\right)\\ {\dot{\kappa }}_{ij}^{2} & =-\epsilon^{2}\left(\kappa_{ij}^{2}+\sin\left(\varphi_{i}^{2}-\varphi_{j}^{2}-\beta \right)\right),\; \end{array}$$ (1)

where the interlayer coupling is modeled by the parameter $\sigma \in R_{\geq 0}$ , and $\alpha^{12}$ and $\alpha^{21}$ are interlayer phase delays. The intrinsic oscillator frequencies are modeled by the parameters $\omega^{1,2}$ , corresponding to the natural metabolic activity. The fixed structure of the organ layer is modeled by the constant all-to-all coupling $a_{ij}^{1}=1$ if i≠j and zero otherwise.

In addition to the adaptive intralayer coupling weights $\kappa_{ij}^{1,2}$ , the intralayer interactions also depend on the phase lag parameters $\alpha^{11}$ and $\alpha^{22}$ , modeling cellular reaction delay. To separate the fast oscillator dynamics from the slower coupling weight dynamics, the adaptation rates $\epsilon^{1,2}$ with $0<\epsilon^{1,2}\ll 1$ are introduced. Since the adaptation of parenchymal cytokine communication is assumed to be slower than the immune counterpart, $\epsilon^{1}\ll \epsilon^{2}\ll 1$ is chosen, which introduces multiple timescales (Sawicki et al., 2022).

Lastly, the most important parameter is β , which controls the adaptivity of the cytokines. Because β has such a strong influence on the model dynamics, it is referred to as the *(biological) age parameter including comorbidities* and summarizing multiple physiological concepts, such as age, inflammatory baselines, adiposity, pre-existing illness, physical inactivity, nutritional influences, and other common risk factors (Berner et al., 2022). Such adaptive couplings have been used to model neural plasticity and learning processes in physiological systems (Jüttner and Martens, 2023). The phase lag parameter β of the adaptation function (also called plasticity rule) plays an essential role in the synchronization process. At a value of $\beta =\frac{\pi }{2}$ , the coupling, and therefore the adaptivity, is at a maximum positive feedback, strengthening the link $\kappa_{ij}$ and encouraging synchronization between oscillators i and j . This maximal connectivity is referred to the *Hebbian Rule* and found in synchronizing systems such as the brain (Berner et al., 2020). For other values $\beta \neq \frac{\pi }{2}$ , the feedback is delayed by a phase lag $\varphi_{i}^{\mu }-\varphi_{j}^{\nu }=\beta -\frac{\pi }{2}$ . For a value of $\beta =-\frac{\pi }{2}$ , we obtain an anti-Hebbian rule, which inhibits synchronization.

A key measure of the system behavior is the derivative of the phases ${\dot{\varphi }}_{i}^{1.2}$ , the instantaneous phase velocities (angular frequencies).

All the system variables and parameters are summarized in Table 1, together with their physiological interpretation.

**TABLE 1:** Summary of the notations used in the physiological network model. Superscripts indicating the layer are omitted for better readability.

Symbol	Name	Physiological meaning
Variable		
$\varphi_{i}$	Phase	Metabolic activity
${\dot{\varphi }}_{i}$	Phase velocity	Frequency of the cellular metabolism
$\kappa_{ij}$	Coupling weight	Cytokine activity
Parameter		
α	Phase lag	Metabolic interaction delay
β	Plasticity rule	Biological age including comorbidities
ω	Natural frequency	Natural cellular metabolism
ϵ	Time-scale ratio	Temporal scale of cytokine activity
$a_{ij}$	Connectivity	Fixed intra-organ cell-to-cell interaction
σ	Interlayer coupling	Interaction through the basal lamina
Measure		
s	Standard deviation of frequency (see Section 2.4)	Pathogenicity (parenchymal layer) Activation (immune layer)

**FIGURE 3:**
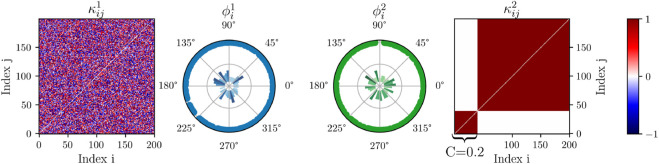
Initialization of the model used for the numerical integration. The leftmost and rightmost panels show the color-coded initial coupling matrices $\kappa_{ij}^{1}$ of the parenchymal layer and $\kappa_{ij}^{2}$ of the immune layer; the two center panels show an exemplary initial random phase distribution of $\varphi_{i}^{1,2}$ in a polar diagram. To model sepsis (Berner et al., 2022), for the immune layer $\left(\kappa_{ij}^{2}\right)$ , an initial cytokine activation described by a two-cluster state is chosen: a smaller cluster of size CN=40 with C=0.2 and a bigger cluster of 160 cells.

The system evolution depends on the choice of initial coupling weights $\kappa_{ij}^{1,2}\left(t=0\right)\in \left[-1,+1\right]$ and phases $\varphi_{i}^{1,2}\left(t=0\right)$ , which are typically drawn from a uniform random distribution over $\left.\left[0,2\pi \right.\right)$ , see Figure 3. Each parameter set is integrated for an ensemble of M=50 random initializations.

Typically, in adaptively coupled oscillators, one can observe several distinct system states that are neither fully synchronized nor desynchronized, such as phase and frequency clusters, chimera states, and splay states (Kasatkin et al., 2017; Berner et al., 2019a; Berner et al., 2019b; Berner et al., 2020; Berner et al., 2023). The emergence of these states depends on the choice of the phase-lag parameters α and β and the interlayer coupling strength σ .

In the multifrequency cluster state, the oscillators do not completely synchronize, but several groups (clusters) of oscillators can form, where the members of each group share a common frequency, but the frequencies of different clusters are different. For in-phase synchronization, the groups additionally synchronize their phase.

The metabolic cell activity is modeled by the phase velocities ${\dot{\varphi }}_{i}$ of the oscillators. The faster the phase velocity, the faster is the metabolism. The members i,j of each layer are adaptively coupled by $\kappa_{ij}^{1,2}$ , and these couplings represent the activity of cytokine mediation. Small absolute coupling values indicate a low communication via cytokines, and it increases with larger coupling strength.

Chimera states, a particular type of partial synchronization, occur when only a subset of oscillators synchronizes in phase and frequency, while others remain desynchronized, induced by spontaneous symmetry breaking. In splay states, all oscillators synchronize their frequencies but not their phases; instead, they are uniformly distributed around the unit circle (Berner et al., 2020).

### 2.3 Pathological states

A biological organism, such as the human body, can be regarded as a self-regulating system that, under healthy conditions, maintains a homeostatic state (Honan and Chen, 2021). Homeostasis refers to a balanced dynamic equilibrium in which the physiological subsystems continuously interact to sustain stability despite external perturbations. In the model, homeostasis is represented by full synchronization of both layers in the duplex network. Pathological states, in contrast, are modeled by the breakdown of synchronicity and the formation of multifrequency clusters in the parenchymal layer, i.e., loss of homeostatic balance. In the model, two clusters form, where the pathological cluster exhibits increased frequency. This aligns with the medical observation that pathological parenchymal cells adopt a less efficient anaerobic glycosis-based metabolism, forcing them to increase their metabolic activity to meet the energy demand (Hanahan and Weinberg, 2011). The healthy frequency-synchronized state may be either phase-synchronized (resilient) or a splay-state (vulnerable), corresponding to weaker coherence (Berner et al., 2022). It is important to note that the temporal evolution of the model trajectories does not directly translate to the evolution of a patient’s state. Instead, only the asymptotic state of desynchronization of the parenchymal layer can be set into correspondence to a patient’s organ functionality.

### 2.4 Synchronicity measures

The instantaneous *mean phase velocity* is calculated as shown in Equation 2:

$${\bar{\omega }}^{1,2}\left(t\right)=\frac{1}{N}\overset{N}{\underset{j}{\sum }}{\dot{\varphi }}_{j}^{1,2}\left(t\right).$$ (2)

The standard deviation of the mean phase velocity is shown in Equation 3:

$$\sigma_{\chi }\left({\bar{\omega }}^{1,2},t\right)=\sqrt{\frac{1}{N}\overset{N}{\underset{j}{\sum }}{\left({\dot{\varphi }}_{j}^{1,2}\left(t\right)-{\bar{\omega }}^{1,2}\left(t\right)\right)}^{2}},$$ (3)

where $\sigma_{\chi }=0$ indicates full frequency-synchronization and increasing values indicate desynchronization or multifrequency clustering. Finally, averaging over m=1,…,M simulations with random initial conditions, the *ensemble-averaged standard deviation of the mean phase velocity* is shown in Equation 4:

$$s^{1,2}\left(t\right)=\frac{1}{M}\overset{M}{\underset{m}{\sum }}\sigma_{\chi }\left({\bar{\omega }}_{m}^{1,2},t\right).$$ (4)

Berner et al. (2022) numerically showed that the quantities $s^{1,2}$ are proportional to the fraction of ensemble members that exhibit frequency clustering. Therefore, this can be used as a measure of the pathological state. The larger $s^{1}$ is, the higher is the risk of multiple organ failure.

### 2.5 Simulation results

The original findings of Berner et al. (2022) identify β , the biological age parameter including comorbidities, and σ , the interlayer coupling strength which models the cytokine activity, as important parameters to understand the underlying mechanisms of sepsis progression. In the following subsection, simulation results are presented. Details on the implementation of the numerical integration can be found in the Supplementary Material.

**FIGURE 4:**
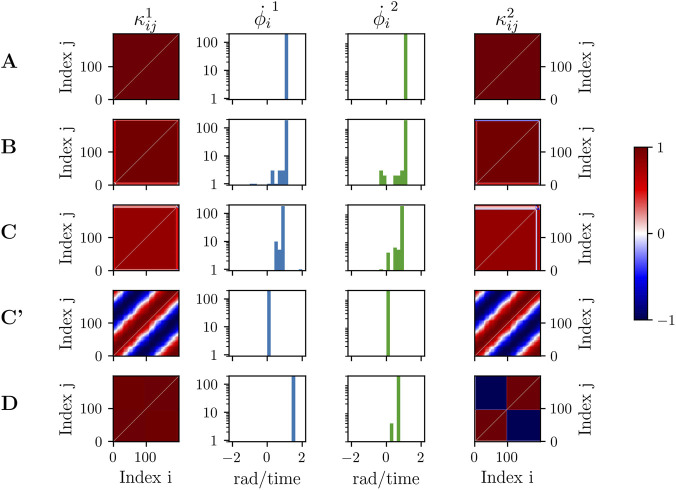
Snapshots of coupling matrices $\kappa_{ij}^{1,2}$ (columns 1 and 4) and instantaneous phase velocities ${\dot{\varphi }}_{i}^{1,2}$ (columns 2 and 3) for different parameters β,σ : healthy parenchymal states without clusters **(A,C’,D)** and a pathological parenchymal state with frequency clusters **(B,C)**. The values of β,σ are indicated as white dots in Figure 5. Configuration C’ corresponds to a vulnerable state because of uniformly distributed phases (splay state). In contrast, **(D)** is regarded as resilient since the phases are fully synchronized. Within each layer μ , the nodes are sorted first by $\left\langle {\dot{\varphi }}_{j}^{1}\right\rangle$ and then by $\varphi_{j}^{1}$ . Parameters: **(A)** β=0.5π,σ=1 , **(B)** β=0.58π,σ=1 , **(C,C’)** β=0.7π,σ=1 , and **(D)** β=0.5π,σ=0.2 . Other parameters are chosen as in Supplementary Table S1 of the Supplementary Material, which also provides further implementation details.

In Figure 4, snapshots of the system variables are shown for different parameter values β and σ . The snapshots are taken at time $T_{\text{sim}}=2000$ and show the stationary values.

In Figure 4, the left-most column depicts the coupling matrices for the organ layer $K^{1}$ , followed by two columns showing the instantaneous phase velocities ${\dot{\varphi }}_{i}^{1,2}$ . The right-most column shows the coupling matrix for the immune layer $K^{2}$ . Rows C and C’ share the same parameters but are different realizations of random initial conditions.

Figure 4A is fully synchronized in frequency and phase and represents a healthy state. The coherence is also visualized in the homogeneous coupling matrices. Panels B and C, in contrast, indicate a pathological state; here, the frequencies split into two distinct clusters, a small and a large, which are also visible in the inhomogeneity of the coupling matrices. Panel C’, although it has the same parameters as C, shows frequency synchronization and hence represents a healthy state; however, it can be considered vulnerable since the phases are uniformly distributed in the $\left.\left[0,2\pi \right.\right)$ interval. Coupling matrices for C’ show the characteristic patterns of splay states. Finally, panel D shows a resilient healthy state, where the frequencies are synchronized and in-phase in the organ layer, while the immune layer remains activated after the initial perturbation and exhibits a small cluster of deviating frequencies and anti-phase synchronization shown in the coupling matrix. This state demonstrates a high degree of parenchymal resilience to the persistent activation of the immune layer.

**FIGURE 5:**
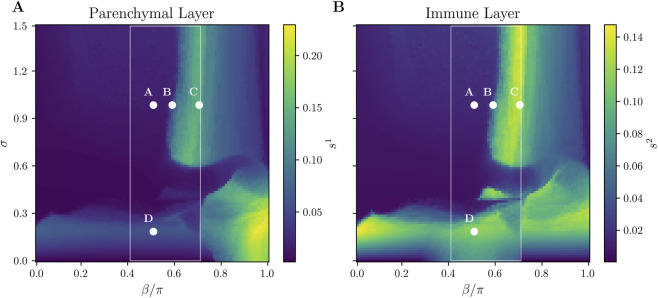
Synchronicity measures $s^{1}$ of the parenchymal layer shown in **(A)** and $s^{2}$ of the immune layer in **(B)**, depending on parameters β and σ . Letters A, B, C, and D indicate the parameter values used in Figure 4. Vertical white lines mark the parameter range to which previous work by Berner et al. (2022) was confined.

To elaborate the dependence of the states upon β and σ , a grid of (β,σ) values was simulated, in the interval $\beta \in \left[0,1\right]$ with a grid resolution of 0.01 and $\sigma \in \left[0,1.5\right]$ with a resolution of 0.015, creating a grid of 10,000 points. The synchronicity measure is $s^{1,2}$ , as shown in Figure 5, in the (β,σ) parameter plane for both layers, where brighter colors indicate a greater risk of organ failure or dysregulated immune response, respectively. The parameters used in panels A, B, C, and D of Figure 4 are indicated as white dots.

It can be observed that smaller values of β<0.55π correspond to stronger synchronization, which is in line with the medical interpretation of β as biological age. For small values of σ<0.5 , the behavior significantly differs between immune and organ layer. The immune layer always shows strong activation (dysregulated immune response), even though the organ layer shows frequency desynchronization only for larger β>0.7π .

## 3 Latent Dynamics Model

The *Latent Dynamics Model* proposed here connects mechanistic modeling and data-driven learning by embedding physical priors from the physiological network model within a neural architecture, following the paradigm of physics-informed machine learning (Raissi et al., 2019). To connect these two worlds, high-dimensional clinical observations are projected into the low-dimensional physical parameter space of the physiological network model, a form of structured representation learning (Bengio et al., 2012), in which latent coordinates carry explicit physiological meaning. Ultimately, the training objectives then serve as a bridge, aligning data-driven flexibility of neural networks with the mechanistic structure imposed by the physiological network model, learning to map patient representations encoded as electronic health records to positions in the physiologically meaningful latent space. This way, model-based explainability will be achieved that goes beyond data-based determination of a sepsis score.

### 3.1 Problem definition

Sepsis onset prediction can generally be categorized into two paradigms: *online* prediction, in which newly arriving medical measurements are incorporated into a continuously updated risk estimate, and *offline* prediction, where only information available at a fixed observation time is used to predict the sepsis risk within a prespecified time-horizon T . In this study, we consider the sepsis risk assessment as an online prediction problem.

For each patient i∈{1,…,P} and observation time $t\in \left\{1,\ldots ,T_{i}\right\}$ , let Equation 5,

$$\mu_{1:t}^{\;i}=\left(\mu_{1}^{\;i},\ldots ,\mu_{t}^{\;i}\right),$$ (5)

denote the history of clinical observations up to time t . The D -dimensional feature vector $\mu_{t}^{\;i}\in R^{D}$ corresponds to an electronic health record, aggregating medical measurements and laboratory results of patients. The sepsis onset event at time t is represented by the binary random variable $S_{t}^{\;i}\in \left\{0,1\right\}$ . Conditioned on the observation history, the sepsis onset event is modeled by a neural network with parameters θ , which defines the conditional distribution (Equation 6):

$$S_{t}^{\;i}∼p_{\theta }\;\left(S_{t}^{\;i}|\mu_{1:t}^{\;i}\right).$$ (6)

Following the definition of Sepsis-3 described in Section 2.1, we consider a sepsis onset event $S_{t}^{\;i}$ as a common outcome of acute worsening in organ function $A_{t}^{\;i}\in \left\{0,1\right\}$ and an indicator for suspected infection $I_{t}^{\;i}\in \left\{0,1\right\}$ (Equation 7):

$$S_{t}^{\;i}=A_{t}^{\;i}\cap I_{t}^{\;i}.$$ (7)

In this study, we employ the physiological network model, which assumes an infected patient state, to derive interpretable measures of organ failure. Consequently, the proposed model decomposes into two separate modules: an *infection module* that estimates suspected infection risk and an *organ-dysfunction module* that estimates acute organ failure risk given infection. This decomposition and application of the probability chain rule yields Equation 8:

$$p_{\theta }\;\left(S_{t}^{\;i}|\mu_{1:t}^{\;i}\right)=p_{\theta }\;\left(A_{t}^{\;i}|I_{t}^{\;i}\cap \mu_{1:t}^{\;i}\right)p_{\theta }\;\left(I_{t}^{\;i}|\mu_{1:t}^{\;i}\right).$$ (8)

Unless specified otherwise, the same symbol θ is used to denote the parameters of both modules and the overall model for notational simplicity. Additionally, we omit the patient index i in the following sections.

### 3.2 Infection module

We model the probability of the suspected infection $I_{t}$ as a function of the observation history $\mu_{1:t}$ via a recurrent neural network, as shown in Equation 9:

$$p_{\theta }\;\left(I_{t}|\mu_{1:t}\right)=\text{sig}\left(W^{f}h_{t}^{f}+b^{f}\right),\;\;h_{t}^{f}=f_{\theta }\left(\mu_{t},h_{t-1}^{f}\right),$$ (9)

where sig:R→(0,1) is the sigmoid function, $h_{t}^{f}\in R^{H_{f}}$ is the hidden state of the recurrent network at time t , $f_{\theta }$ is a GRU cell with parameters θ , and $W^{f}\in R^{1\times H_{f}}$ and $b^{f}\in R$ are learnable parameters of the linear output layer. The initial hidden state $h_{0}^{f}$ is shared between all patients and treated as a learnable vector.

To account for temporal uncertainties of the diagnosis, we replace the binary $I_{t}$ indicator with a temporally smoothed surrogate ${\bar{I}}_{t}\in \left[0,1\right]$ that increases linearly from 0.1 in the 48 h preceding the infection onset, reaches maximum at onset with a value of 1, and decays exponentially back to 0.1 afterward in the subsequent 24 h. This smoothed version mimics temporal uncertainty of the diagnosis, for example, due to delayed documentation and treatment effects such as antibiotic half-life.

### 3.3 Organ-dysfunction module

The acute worsening in organ function is defined as a binary variable $A_{t}\in \left\{0,1\right\}$ , indicating an increase in the SOFA score according to the Sepsis-3 criteria. Instead of modeling this outcome directly, we estimate the SOFA score as a continuous variable $O_{t}\in \left[0,24\right]$ in a physiologically interpretable way, leveraging the physiological network model parameter space (β,σ) . In this space, the parenchymal desynchronization measure $s^{1}\left(\beta ,\sigma \right)$ acts as a proxy for organ dysfunction, so that Equation 10 can be obtained:

$${\hat{O}}_{t}\propto s^{1}\left(\beta_{t},\sigma_{t}\right)\left[\right].$$ (10)

Because β and σ are latent model parameters and cannot be derived directly from medical observations, we infer them with neural networks.

To capture acute organ failure, we relax the original Sepsis-3 criterion $O_{t}-O_{t-1}>2$ by introducing learnable shift d∈R and scale m∈R parameters and applying temporal smoothing (Equation 11):

$$p_{\theta }\;\left(A_{t}|I_{t}\cap \mu_{1:t}\right)=\overset{r}{\underset{\tau =0}{\sum }}w_{\tau }\cdot \text{sig}\left(m\left({\hat{O}}_{t-\tau }-{\hat{O}}_{t-\tau -1}-d\right)\right),\;w_{\tau }=\frac{e^{-\alpha \tau }}{\sum_{k=0}^{r}e^{-\alpha k}},$$ (11)

where $r\in N_{>0}$ defines the temporal window and $\alpha \in R_{>0}$ is a learnable decay parameter controlling kernel shape. For t−τ≤0 , we set ${\hat{O}}_{t-\tau }-{\hat{O}}_{t-\tau -1}=0$ . The causal smoothing reflects the clinical observation that organ dysfunction typically precedes documented sepsis onset and that sepsis is a sustained physiological state rather than an instantaneous event.

Prediction is carried out in the physical parameter space of the physiological network model. To this end, the organ-dysfunction module maps patient data to a two-dimensional latent representation $z=\left(z_{1},z_{2}\right)$ , produced by a neural network. These latent coordinates are subsequently transformed into the model parameters (β,σ) via Equation 12:

$$\begin{array}{cc} \beta & =\text{sig}\left(z_{1}\right)\left(\beta_{\max}-\beta_{\min}\right)+\beta_{\min}\\ \sigma & =\text{sig}\left(z_{2}\right)\left(\sigma_{\max}-\sigma_{\min}\right)+\sigma_{\min}, \end{array}$$ (12)

where $\beta_{\min},\beta_{\max}$ and $\sigma_{\min},\sigma_{\max}$ indicate the boundaries of a prespecified region in the parameter space. As new measurements arrive, the patient’s position in this parameter space is updated, generating trajectories through the physical parameter space. The resulting $\left(\beta_{1:t},\sigma_{1:t}\right)$ trajectory is then evaluated via $s^{1}\left(\beta_{1:t},\sigma_{1:t}\right)$ to produce ${\hat{O}}_{1:t}$ , which directly feeds into the acute worsening criterion in Equation 11.

To obtain the latent coordinates $\left(\beta_{t},\sigma_{t}\right)$ that parameterize the physiological network model, we employ a neural encoder-recurrent architecture. Given an initial electronic health record vector $\mu_{1}$ , an encoder network $g_{\theta }$ produces a latent coordinate $z_{1}\in R^{2}$ and a hidden representation $h_{1}^{g}\in R^{H_{g}}$ , as shown in Equation 13:

$$\left(z_{1},h_{1}^{g}\right)=g_{\theta }\left(\mu_{1}\right).$$ (13)

For the subsequent time points, a recurrent neural network $q_{\theta }$ , specifically a GRU, continuously updates the hidden state and latent coordinates based on newly observed electronic health record data, as presented in Equations 14–16:

$$h_{t}^{g}=q_{\theta }\left(\left(\mu_{t},z_{t-1}\right),h_{t-1}^{g}\right),$$ (14)

$$\Delta z_{t}=W^{g}h_{t}^{g},$$ (15)

$$z_{t}=z_{t-1}+\Delta z_{t},$$ (16)

for t=2,…,T , where $W^{g}\in R^{2\times H_{g}}$ is a learned linear projection mapping from $h_{t}^{g}$ to a step in the physical parameter space $\Delta z_{t}$ at time t and $z_{t}$ is the updated latent position via residual addition. Scaling $z_{1:t}$ yields a trajectory $\left(\beta_{1:t},\sigma_{1:t}\right)$ through the physiological parameter space.

### 3.4 Latent lookup

To avoid the computational burden and gradient instabilities of backpropagating through the integration of the coupled differential equations with respect to the parameters (β,σ) , particularly for long simulation horizons and large ensembles, we propose a quantization-based *latent lookup* strategy that approximates the desynchronization dynamics of the physiological network model while preserving differentiability via interpolation (Kidger and Garcia, 2021; Sophiya et al. 2025).

**FIGURE 6:**
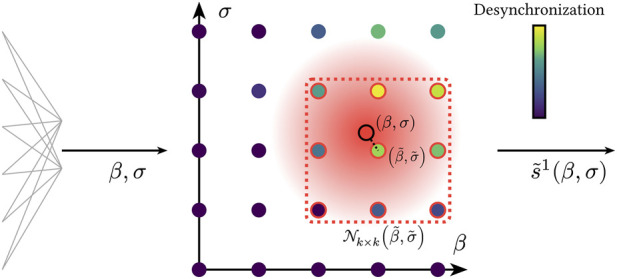
Quantized latent lookup of precomputed synchronization metrics. Similar to Figure 5, grid-point colors represent the amount of desynchronization $s^{1}$ in the parenchymal layer. Neighboring points, the $\left(\beta^{\prime },\sigma^{\prime }\right)\in N_{3\times 3}\left(\tilde{\beta },\tilde{\sigma }\right)$ sub-grid, indicated by the red outlines and the red rectangle around $\left(\tilde{\beta },\tilde{\sigma }\right)$ , are used smoothed using a Gaussian-like kernel, represented by the gradient around the estimated point (β,σ) , allowing continuous interpolation of the parameter space.

For an estimated coordinate in the continuous (β,σ) space, the precomputed desynchronization metrics are interpolated over nearby grid points using a Gaussian-like kernel (see Figure 6). Differentiability is maintained via two mechanisms. First, we associate each parameter pair (β,σ) with its nearest grid point $\left(\tilde{\beta },\tilde{\sigma }\right)$ , and centered around this nearest point, we define a k×k neighborhood, as presented in Equation 17:

$$N_{k\times k}\left(\tilde{\beta },\tilde{\sigma }\right)=\left\{\left(\tilde{\beta }+i\cdot \beta_{\text{step}}\right),\left(\tilde{\sigma }+j\cdot \sigma_{\text{step}}\right)|i,j\in -\frac{k-1}{2},\ldots ,-1,0,1,\ldots ,\frac{k-1}{2}\right\},$$ (17)

where k>1 is odd.

Second, a differentiable softmax interpolation is applied on the synchronicity measure of the K=k×k neighboring grid points, as shown in Equation 18:

$${\tilde{s}}^{1}\left(\beta_{t},\sigma_{t}\right)\left[\right]=\underset{\left(\beta^{\prime },\sigma^{\prime }\right)\in N_{k\times k}\left(\tilde{\beta },\tilde{\sigma }\right)}{\sum }\text{softmax}\;\left(-\frac{\|\left(\beta_{t},\sigma_{t}\right)\left[\right]-\left(\beta^{\prime },\sigma^{\prime }\right){\|}^{2}}{T_{d}}\right)s^{1}\left(\beta^{\prime },\sigma^{\prime }\right).$$ (18)

The learnable temperature $T_{d}\in R_{>0}$ controls the interpolation sharpness: larger values produce broad smoothing, while smaller values concentrate weight on the nearest grid point for precision.

This strategy is closely related to *finite scalar quantization* (Mentzer et al., 2023), as employed in Dreamer-V3 (Hafner et al., 2024), and similarly enables differentiable quantization. The two key distinctions set it apart: latent values are continuously interpolated rather than snapped to the nearest grid point, and the latent coordinates carry explicit semantic meaning, corresponding to physiological parameters β and σ and organ states, whereas in Dreamer-V3, the latent space is semantically unconstrained.

### 3.5 Decoder

To encourage a semantically structured parameter space, a neural decoder module is added at training time as an auxiliary regularization component, attempting to reconstruct observed electronic health record features from the latent representation:

$${\hat{\mu }}_{t}=d_{\theta }\left(\beta_{t},\sigma_{t}\right).$$ (19)

This latent regularization is motivated by *representation learning* (Bengio et al., 2012) and ensures that nearby points in the latent (β,σ) space correspond to physiologically similar patient states. It should help the encoder $g_{\theta }$ learn a meaningful alignment between electronic health record-derived latent-embeddings and the physiological network model landscape.

### 3.6 Training objective

**FIGURE 7:**
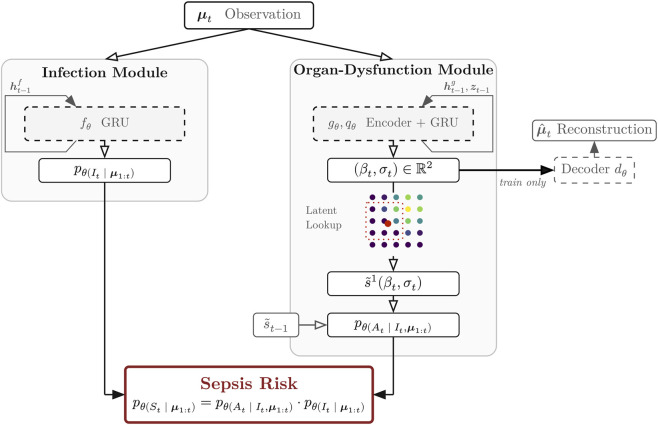
Complete Latent Dynamics Model architecture with three main components. The infection module $f_{\theta }$ and the SOFA module $g_{\theta }$ process electronic health record data $\mu_{t}$ through recurrent networks to estimate the infection level $p_{\theta }\;\left(I_{t}|\mu_{1:t}\right)$ and latent coordinates $z_{t}$ , respectively. The latent coordinates map to organ failure $O_{t}$ , from which acute changes $p_{\theta }\;\left(A_{t}|I_{t}\cap \mu_{1:t}\right)$ are computed using consecutive predictions. The heuristic organ failure risk is assumed to be 0 for the initial time step. The decoder $d_{\theta }$ reconstructs electronic health record features $\mu_{t}$ from latent coordinates, regularizing the parameter space to maintain clinically meaningful structure. Final sepsis risk $p_{\theta }\;\left(S_{t}|\mu_{1:t}\right)$ combines infection and acute change signals. Schematic representations of the two modules are shown in Supplementary Figures S5, S6.

At training time, all modules, namely, the infection module $f_{\theta }$ , the organ-dysfunction module $g_{\theta },q_{\theta }$ , and the decoder $d_{\theta }$ , are trained jointly. The complete architecture of the Latent Dynamics Model can be seen in Figure 7. The training objective combines a primary loss for sepsis onset prediction with auxiliary losses that guide the infection and organ-dysfunction modules and regularize the latent representation.

Starting with the primary training objective, which aligns the predicted risk score $p_{\theta }\;\left(S_{t}|\mu_{1:t}\right)=p_{\theta }\;\left(A_{t}|I_{t}\cap \mu_{1:t}\right)p_{\theta }\;\left(I_{t}|\mu_{1:t}\right)$ with ground truth sepsis labels via sample-averaged binary cross entropy over mini-batches of size B , we can obtain Equation 20:

$$L_{\text{sepsis}}=-\frac{1}{B}\overset{B}{\underset{i=1}{\sum }}\frac{1}{T_{i}}\overset{T_{i}}{\underset{t=1}{\sum }}\left[S_{t}^{i}\log\left(p_{\theta }\;\left(S_{t}^{i}|\mu_{1:t}^{\;i}\right)\right)+\left(1-S_{t}^{i}\right)\log\left(1-p_{\theta }\;\left(S_{t}^{i}|\mu_{1:t}^{\;i}\right)\right)\right].$$ (20)

To explicitly supervise the infection module, we introduce an auxiliary binary cross entropy loss (Equation 21):

$$L_{\text{inf}}=-\frac{1}{B}\overset{B}{\underset{i=1}{\sum }}\frac{1}{T_{i}}\overset{T_{i}}{\underset{t=1}{\sum }}\left[{\bar{I}}_{t}^{i}\log\left(p_{\theta }\;\left(I_{t}^{i}|\mu_{1:t}^{i}\right)\right)+\left(1-{\bar{I}}_{t}^{i}\right)\log\left(1-p_{\theta }\;\left(I_{t}^{i}|\mu_{1:t}^{i}\right)\right)\right].$$ (21)

To fit the organ-dysfunction module-encoding functions, the placement of parameter coordinates (β,σ) is guided by a supervision signal through a mean-squared-error loss (Equation 22):

$$L_{\text{sofa}}=\frac{1}{B}\overset{B}{\underset{i=1}{\sum }}\frac{1}{T_{i}}\overset{T_{i}}{\underset{t=1}{\sum }}w_{O}\cdot {\left(\frac{O_{t}^{i}}{24}-\frac{{\tilde{s}}_{i}^{1}\left(\beta_{t},\sigma_{t}\right)}{s_{\text{max}}^{1}}\right)}^{2},$$ (22)

where the class-balancing weight is presented in Equation 23:

$$w_{O}=\log\left(1+f_{O}^{-1}\right),$$ (23)

with $f_{O}$ being the relative frequency of SOFA scores O . This inverse frequency weighting upweights rare high SOFA scores. It is also noticed that both parts, i.e., the continuous approximation (given by the desynchronicity) and ground truth, are scaled to the interval $\left[0,1\right]$ .

Two additional auxiliary losses are used to regularize the latent representations, as shown in Equation 24:

$$L_{\text{spread}}=-\log\left(\det\left(\text{Cov}\left(\hat{Z}\right)\right)\right),$$ (24)

where $\hat{Z}\in R^{2\times \sum_{i=1}^{B}T_{i}}$ collects all predicted latent coordinates $z_{t}^{i}$ across the mini-batch, i.e., the patients i=1,…,B and their corresponding time steps $t=1,\ldots ,T_{i}$ . This loss maximizes the *generalized variance* (Carroll and Green, 1997) of the latent dimensions β and σ , encouraging the model to spread predictions across the parameter space rather than collapsing to a degenerate distribution.

To keep the predicted latent points inside a predefined area, they will be discouraged to move too close to the edges (Equation 25):

$$L_{\text{boundary}}=\text{ReLU}\left(f-\text{sig}\left(z_{t}\right)\right)+\text{ReLU}\left(\text{sig}\left(z_{t}\right)-\left(1-f\right)\right),$$ (25)

where $f\in \left.\left[0,0.5\right.\right)$ sets a boundary threshold as a fraction of the space, creating a penalty buffer that discourages latent variables from entering the outer f -percent of the space near the edges.

Lastly, the decoder is trained using a mean-squared-error-supervised loss, relating the ground truth electronic health record to the reconstruction (Equation 26):

$$L_{\text{dec}}=\frac{1}{B}\overset{B}{\underset{i=1}{\sum }}\frac{1}{T_{i}}\overset{T_{i}}{\underset{t=1}{\sum }}{\left(\mu_{t}^{i}-{\hat{\mu }}_{t}^{i}\right)}^{2}.$$ (26)

The total loss combines all objectives as a weighted sum (Equation 27):

$$\begin{array}{llllll} L_{\text{total}}=\; & \lambda_{\text{sepsis}}L_{\text{sepsis}} & +\; & \lambda_{\text{inf}}L_{\text{inf}} & +\; & \lambda_{\text{sofa}}L_{\text{sofa}}\\ & +\lambda_{\text{dec}}L_{\text{dec}} & +\; & \lambda_{\text{spread}}L_{\text{spread}} & +\; & \lambda_{\text{boundary}}L_{\text{boundary}}. \end{array}$$ (27)

### 3.7 Clinical application

The Latent Dynamics Model operates as a continuous monitoring system throughout a patient’s stay in an intensive care unit. Upon admission, the initial electronic health record $\mu_{1}$ is processed by both modules, providing immediate baselines of infection risk $p_{\theta }\left(I_{1}|\mu_{1}\right)$ and an indication of organ functionality through the synchronicity measure $s^{1}\left(\beta_{1},\sigma_{1}\right)$ . Subsequently, each new electronic health record $\mu_{t}$ , arriving either event-triggered or at regular hourly intervals, is processed by the recurrent modules to update $p_{\theta }\;\left(I_{t}|\mu_{1:t}\right)$ and $\left(\beta_{t},\sigma_{t}\right)$ . Acute organ failure risk $p_{\theta }\;\left(A_{t}|I_{t}\cap \mu_{1:t}\right)$ and sepsis risk $p_{\theta }\;\left(S_{t}|\mu_{1:t}\right)$ are then derived from the evolving latent trajectory $\left(\beta_{t:1},\sigma_{t:1}\right)$ , and a sepsis alert is triggered when $p_{\theta }\;\left(S_{t}|\mu_{1:t}\right)$ exceeds a predefined threshold. This continues until the patient is discharged.

A key advantage of the proposed decomposition is that the model produces four clinically interpretable outputs at each time step, as summarized in Table 2.

**TABLE 2:** Outputs of the Latent Dynamics Model at each time step t , their value ranges, and clinical interpretation.

Symbol	Range	Clinical meaning
$p_{\theta }\;\left(I_{t}|\mu_{1:t}\right)$	(0,1)	Infection likelihood
$\left(\Delta \beta_{t},\;\Delta \sigma_{t}\right)$	$R^{2}$	Latent trajectory step (physiological stability)
${\tilde{s}}^{1}\left(\beta_{t},\sigma_{t}\right)$	[0,1]	Organ system desynchronization (SOFA proxy)
$p_{\theta }\;\left(A_{t}|I_{t}\cap \mu_{1:t}\right)$	(0,1)	Acute organ failure risk (recent worsening)
$p_{\theta }\;\left(S_{t}|\mu_{1:t}\right)$	(0,1)	Overall sepsis risk (primary alert signal)

Rather than issuing a single opaque risk score, the model allows clinicians to distinguish whether an elevated sepsis risk is driven primarily by suspected infection, acute organ deterioration, or both. This transparency supports more informed clinical decision-making, for example, by differentiating patients who require immediate antimicrobial treatment from those whose risk stems predominantly from organ failure. The latent trajectory $\left(\beta_{1:t},\sigma_{1:t}\right)$ further provides an interpretable record of the patient’s physiological evolution through parameter space during the intensive care unit stay. Additionally, the speed of movement through the latent space, $\left(\left\|\Delta \beta_{t}\|,\|\Delta \sigma_{t}\right\|\right)$ , provides a natural indicator of physiological stability.

## 4 Training with intensive care unit data

We trained and evaluated the proposed Latent Dynamics Model on real-world ICU data. The following subsections detail the dataset, preprocessing, and training procedure.

### 4.1 Data source, cohort, and preprocessing

Our study relies exclusively on the MIMIC-IV database (version 2.2) (Johnson et al., 2023b). The MIMIC database series contains data on routine clinical care, including patient measurements, medication orders, diagnoses, procedures, treatments, and free-text clinical notes. Although models trained on a single dataset may exhibit limited external generalization, the MIMIC database remains the most widely used open clinical dataset for developing and benchmarking sepsis onset prediction systems (Bomrah et al., 2024; Rockenschaub et al., 2023).

The proposed methods for predicting sepsis often have the following shortcomings: (1) incomparability and (2) limited reproducibility due to undisclosed and heterogeneous data processing, task definition, and implementation. To address this, we adopt the YAIB framework (van de Water et al., 2024) for all steps of cohort and task definition, sepsis labeling, feature extraction, and data preprocessing. YAIB standardizes retrospective intensive care unit studies across publicly available datasets, enabling direct comparison with benchmark results for common intensive care unit prediction tasks, including online sepsis onset prediction. According to the definition of YAIB, the primary prediction target is to detect the sepsis onset time within 6 h, where the sepsis labeling closely follows the Sepsis-3 criteria. Although the MIMIC databases are limited to intensive care unit patients, requiring the model to distinguish severely ill patients with sepsis from those without, the proposed method could, in principle, also be extended to differentiate healthy individuals.

The base cohort (N = 73,181) is filtered by removing patient stays with total length of stay < 6 h (N = 1,004), < 4 h without any measurement (N = 50), ≥12 hours between any measurements, and sepsis onset time < 6 h after admission (N = 8,537), resulting in a final cohort of 63,425 patients^1^, of which 3,320 (5.2%) met the sepsis criteria. Sepsis-positive patients show notably higher disease severity. The median maximum SOFA score is 5.0 compared to 4.0 in sepsis-negative patients, and hospital mortality is substantially higher (26.5% vs. 6.6%). Septic patients also have longer hospital stays (median 335.1 h vs. 150.3 h). The median time to sepsis onset is 13 h after ICU admission, with a 25th–75th percentile interquartile range of 8–34 h.

Each electronic health record includes 52 input features, with 4 static variables (age, height, and weight at admission, as well as sex) and 48 dynamic time-series variables. Dynamic variables combine 7 vital signs (e.g., heart rate, blood pressure, and body temperature, among others) and 39 laboratory tests (e.g., blood sugar level and kidney function, among others), and 2 additional measurements (fraction of inspired oxygen and urine output). Further cohort descriptions and an exhaustive listing of the input features, including missingness statistics, can be found in our Supplementary Figure S3; Supplementary Tables S2–S4.

Training employs five repetitions of 5-fold cross validation, resulting in 25 independent training runs, called splits. For each run, the cohort was partitioned at the patient level using a stratified split, with a 80/10/10 ratio for training, validation, and test sets, respectively, yielding N=50,740/6,343/6,342 patients. Splitting was stratified by sepsis status to maintain the 5.2% prevalence ratio across all sets.

As the preprocessing step, also adopted from YAIB, the data are resampled to an hourly resolution, where missing points for dynamic variables were forward-filled using the last known value of the same intensive care unit stay. Missing values without any prior measurement, the training cohort mean is used as fill value instead. Lastly, the input data are augmented by a binary indicator that distinguishes between actual measurements and imputed values, effectively doubling the number of input features. Lastly, all continuous variables have been standardized to zero mean and unit variance.

### 4.2 Training details

Hyperparameters were manually tuned once prior to cross-validation. Automated optimization was avoided because the loss function jointly optimizes predictive performance and latent-space interpretability, and reducing these objectives to a single scalar metric could bias optimization toward predictive accuracy at the cost of degraded latent representations, defeating the core purpose of embedding the physiological network model.

The physical parameter space was quantized to a 60×100 grid over $\beta \in \left[0.4\pi ,0.7\pi \right]$ and $\sigma \in \left[0.0,1.5\right]$ , covering the physiologically plausible regime of synchronization dynamics observed in prior physiological network model simulations (Berner et al., 2022) The differential lookup used a 7×7 neighborhood softmax interpolation (K=49) .

The objective component weights are as follows: $\lambda_{\text{inf}}=1.0$ for the infection module specific loss, $\lambda_{\text{sofa}}=2\times 10^{3}$ for the organ-dysfunction module, with the auxiliary $\lambda_{\text{spread}}=6\times 10^{-3}$ spreading term, $\lambda_{\text{boundary}}=30.0$ with a boundary margin f=0.1 , the primary objective $\lambda_{\text{sepsis}}=600.0$ , and $\lambda_{\text{dec}}=2.5$ for the decoder. Lastly, τ=12 , controlling the radius of the causal smoothing. Learnable parameters were initialized as follows: the detection threshold d=0.04 and sharpness m=50 , causal smoothing decay α=0.7 , and the initial lookup interpolation temperature $T_{d}=0.05$ .

All modules were jointly optimized using an AdamW optimizer (Loshchilov and Hutter, 2019), with a mini-batch size of 64. The first epoch serves as a warm-up phase, where the learning rate increases linearly from 0.0 to $5\times 10^{-5}$ . Subsequently, a constant learning rate is maintained for all remaining epochs. For the optimizer configuration, a weight decay of λ=0.2 and momentum parameters $\beta_{1}=0.9$ and $\beta_{2}=0.999$ are chosen. To prevent exploding gradients, global gradient clipping with a maximum norm of c=1.0 was applied. Details on the encoder and decoder architectures $g_{\theta },q_{\theta },d_{\theta }$ , the MLP replacement, and learnable parameter counts of each module are provided in Supplementary Figures S4–S7; Supplementary Table S5.

Training was carried out on a consumer laptop GPU (NVIDIA RTX 500 Ada Generation with 4 GB of VRAM) using 32-bit floating-point precision. Training lasted a maximum of 1,000 epochs, and to prevent overfitting, early stopping was employed, where training stops after 10 consecutive epochs in which neither AUROC nor AUPRC improved on the validation set. A typical training run to convergence required approximately 40 min, with early stopping occurring between epoch 50 and 125.

For each run, the model checkpoint was selected at the epoch corresponding to the geometric mean of the validation AUROC peak and AUPRC peak epochs. This heuristic balances optimization for both metrics while avoiding bias toward a single evaluation criterion. Subsequently, this model is tested against the held-out test set.

### 4.3 Evaluation

To quantitatively assess the sepsis onset prediction performance, we rely on the AUROC and AUPRC (McDermott et al., 2025), averaged over the different splits. Both are commonly reported metrics in the sepsis onset prediction literature by Moor et al. (2021). The AUROC measures a classifier’s ability to discriminate between septic and non-septic patients across all classification thresholds, while the AUPRC is particularly informative in the context of class imbalance, a characteristic feature of sepsis datasets, where positive cases are relatively rare. The same metrics are used to evaluate the prediction performance on the auxiliary suspected infection and acute organ failure labels. For organ-dysfunction prediction, performance is additionally evaluated using the root-mean-squared error (RMSE) of the SOFA score:

$$\text{RMSE}=\sqrt{\frac{1}{P}\overset{P}{\underset{i=1}{\sum }}\frac{1}{T_{i}}\overset{T_{i}}{\underset{t=1}{\sum }}{\left(O_{t}^{i}-{\hat{O}}_{t}^{i}\right)}^{2}},$$

where ${\hat{O}}_{t}^{i}$ is the predicted and $O_{t}^{i}$ the ground truth SOFA score for patient i at time t , averaged over time and patients.

Beyond quantitative metrics, we qualitatively investigate, for a single split, whether the Latent Dynamics Model generates plausible latent trajectories at both a systemic and an individual patient level.

To put our results into context, we consider the following methods for comparison, following the benchmarking setup of YAIB (van de Water et al., 2024). Classical machine-learning approaches include regularized logistic regression and light gradient boosted machines (Ke et al., 2017). For deep-learning baselines, four sequence modeling architectures are included: GRU (Cho et al., 2014), long short-term memory (Hochreiter and Schmidhuber, 1997), temporal convolutional network (Bai et al., 2018), and transformer Vaswani et al. (2017). As an additional baseline, we consider a model identical to the Latent Dynamics Model but replace the lookup mechanism with a small randomly initialized but learnable multi-layer perceptron (MLP). This helps us understand whether the latent space given by the physiological network model provides a useful prior.

All baselines were trained on the same data and task definition and evaluated using the same cross-validation strategy as our approach, ensuring a fair comparison. In contrast to our method, the YAIB benchmarks were obtained using Bayesian hyperparameter optimization applied independently for each model and split. As specific hyperparameter configurations are not reported by YAIB, we refer the reader to the original framework for implementation details (van de Water et al., 2024). To assess whether observed performance differences are statistically significant, we apply a two-sided Welch’s t -test (Welch, 1947), with Holm–Bonferroni correction (Holm, 1979) applied across all YAIB comparisons, without assuming equal variance.

## 5 Results

**FIGURE 8:**
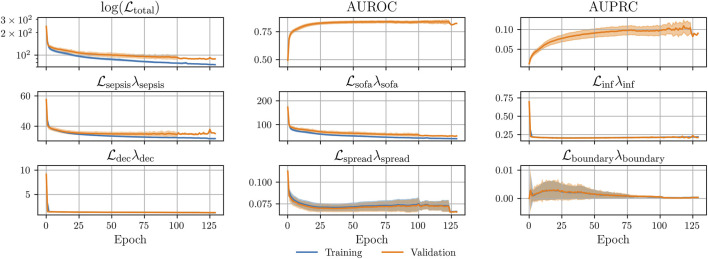
Training and validation curves (mean and standard deviation) of the Latent Dynamics Model showing the evolution of the total loss, task metrics AUROC and AUPRC, and all individual loss components. The plots illustrate stable multi-objective convergence, early alignment of infection $f_{\theta }$ and SOFA $g_{\theta }$ submodules, and gradual refinement of the final sepsis risk prediction.

To confirm stable training dynamics despite the potential for objective competition within the multi-objective loss, we plot the progression of the total loss $L_{\text{total}}$ , each loss component, and the validation AUROC and AUPRC metrics in Figure 8. Each curve represents the mean value, with shaded areas indicating one standard deviation across the 25 splits. All components, except $L_{\text{boundary}}$ , exhibit a rapid decrease in the first five epochs, followed by gradual refinement, with training and validation curves remaining closely aligned throughout.

The loss components associated with the infection and organ failure modules decrease steeply in the early epochs, consistent with the auxiliary tasks being resolved relatively quickly. The sepsis onset prediction loss follows a smoother, more gradual descent, suggesting that the final risk prediction continues to refine throughout training as the latent representation improves. This staged convergence is consistent with the design intent of the loss function, where auxiliary supervision guides representation learning. Correspondingly, validation AUROC increases from near-random performance (0.5) to approximately 0.84, and AUPRC shows a similar upward trend, particularly relevant given the strong class imbalance in the data.

### 5.1 Sepsis onset prediction results

Table 3 compares the Latent Dynamics Model against the considered baseline methods. The Latent Dynamics Model achieves the highest mean AUROC and AUPRC among the evaluated models, although with higher variance across runs across all 25 splits. To assess statistical significance, we apply a two-sided Welch’s t -test, which does not assume equal variance across models, which is appropriate given the observed differences in spread. As shown in Table 3, statistically significant improvements are observed across all baselines in both metrics. The most substantial gains are observed relative to the classical baselines, reflecting their limited capacity to capture temporal dynamics. Among the deep-learning baselines, the improvements remain significant, even against the strongest baseline, GRU; significance is confirmed for both AUROC (t(30.40)=2.78,p<0.01) and AUPRC (t(29.49)=4.37,p<0.001) ), indicating improvements in discriminative power and precision–recall balance. Overall, the results indicate that incorporating physiological network model-based latent structure does not compromise predictive performance while providing a physiologically interpretable latent representation.

**TABLE 3:** Predictive performance of baseline models compared to that of the proposed Latent Dynamics Model, in terms of AUROC×100↑ and AUPRC×100↑ , within one standard deviation (±) . Each metric is calculated over five iterations of five-fold cross-validation, i.e. 25 splits. Statistical significance of differences is assessed via a two-sided Welch’s t -test, reported as t -statistic and p -value, with Holm–Bonferroni correction applied across all 12 YAIB comparisons to control the family-wise error rate.

Model	AUROC (±)	t	p	AUPRC (±)	t	p
YAIB van de Water et al. (2024)						
Regularized logistic regression	77.1 ( ± 0.4)	38.48	< 0.001	4.6 ( ± 0.1)	29.95	< 0.001
Light gradient boosting	77.5 ( ± 0.3)	37.92	< 0.001	5.9 ( ± 0.2)	22.21	< 0.001
Transformer	80.0 ( ± 0.8)	17.88	< 0.001	6.6 ( ± 0.2)	18.33	< 0.001
Long short-term memory	82.0 ( ± 0.3)	11.99	< 0.001	8.0 ( ± 0.2)	10.59	< 0.001
Temporal convolutional network	82.7 ( ± 0.3)	7.96	< 0.001	8.8 ( ± 0.2)	6.17	< 0.001
GRU	**83.6** ( ± 0.3)	2.78	0.009	**9.1** ( ± 0.3)	4.37	< 0.001
This work						
Latent dynamics model w/ MLP	84.07 ( ± 0.7)	0.07	1.0	9.89 ( ± 0.9)	0.06	1.0
Latent dynamics model	84.08 (±0.8)	—	—	9.91 (±0.9)	—	—

The bold values indicate the best performing model for each family.

The Latent Dynamics Model with MLP replacement performs identically; there is no evidence that predictive performance is uniquely attributable to the physiological prior. To further characterize the source of predictive signal, we report an inference-time decomposition of the trained Latent Dynamics Model into its modules. Using the jointly trained checkpoints without retraining, we evaluate by fixing the complementary branch to a neutral value (effectively removing its contribution to the multiplicative interaction term), meaning to assess the infection module $p_{\theta }\;\left(I_{t}|\mu_{1:t}\right)\cdot 1$ and for the organ-dysfunction module $1\cdot p_{\theta }\;\left(A_{t}|I_{t}\cap \mu_{1:t}\right)$ as predictors of sepsis onset $S_{t}$ .

Under this decomposition, the infection module achieves 83.50±0.89 AUROC and 9.68±0.97 AUPRC, which is close to the performance of the full joint model (84.01 AUROC). In contrast, the organ-dysfunction module alone achieves 58.72±2.82 AUROC and 1.88±0.17 AUPRC, substantially below the full model and other baselines. These results provide an indication of how predictive information is distributed across the learned modules under the trained model parameters. In particular, the infection module carries most of the discriminative signal for the evaluated task, while the organ-dysfunction module on its own has limited predictive capacity. The joint model slightly improves over the infection module, suggesting a modest additional contribution from the coupled representation. Taken together, these results suggest that the value of the organ-dysfunction module lies primarily in its interpretable trajectory representation rather than in discriminative lift, a point elaborated in Section 5.3.

### 5.2 Intermediate targets: infection and organ dysfunction

**FIGURE 9:**
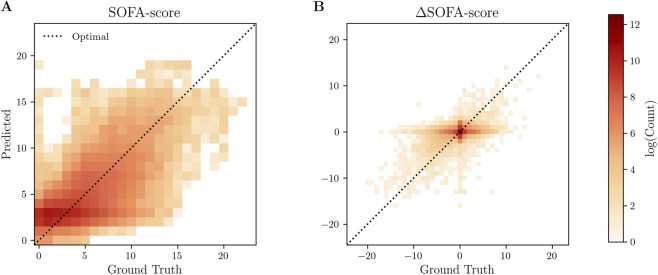
Density plots comparing ground truth and predicted values. **(A)** The SOFA score and **(B)** the immediate SOFA change ( Δ SOFA). The model captures the overall SOFA severity distribution and its temporal changes. Color indicates log sample density.

For the following results, only a single trained model is investigated. Here, we choose the model closest to the mean AUROC and AUPRC over all cross-validation splits. The entire operator-receiving and precision–recall curves for this model are shown in Supplementary Figure S8 of the Supplementary Material. Figure 9 shows sample-density plots comparing predicted and ground-truth values, demonstrating that the model captures both the magnitude and temporal dynamics of organ dysfunction and infection. For each variable, a diagonal line corresponds to optimal prediction performance.

Predictions for the SOFA score magnitude follow the diagonal trend across the full severity range, achieving an RMSE of 2.21. This suggests that the model preserves the ordinal structure of organ failure rather than collapsing toward the mean, although predictions remain most concentrated around moderate severity levels. For the change in the SOFA score ( Δ SOFA between consecutive time steps), the distribution is strongly centered around zero for both ground truth and predictions, reflecting that most time steps do not involve acute changes. The model captures both the concentration and the spread toward positive and negative deviations, indicating that it learns not only absolute severity but also the direction and magnitude of temporal organ deterioration or recovery, where deterioration is defined as any increase in the SOFA score. However, some residual misalignment remains, particularly in cases where the ground truth increases, while the model predicts a decrease, and *vice versa*.

The Δ SOFA-based proxy for acute organ failure reaches an AUROC of 66.01 and an AUPRC of 9.95. For the infection-related outputs, the suspected infection submodule achieves an AUROC of 72.85 and an AUPRC of 20.03. These results confirm that the auxiliary supervision targets are learned meaningfully, providing structured guidance to the latent representation during training.

The lower Sub-module scores are constrained by the Sepsis-3 definition: infection signals are broad and flexible, whereas SOFA increases are sparse, discrete events that strictly penalize timing offsets. The joint prediction achieves superior performance by leveraging a complementary error structure and applying the causal smoothing strategy to stabilize the SOFA signal.

### 5.3 Interpretability via latent trajectories

**FIGURE 10:**
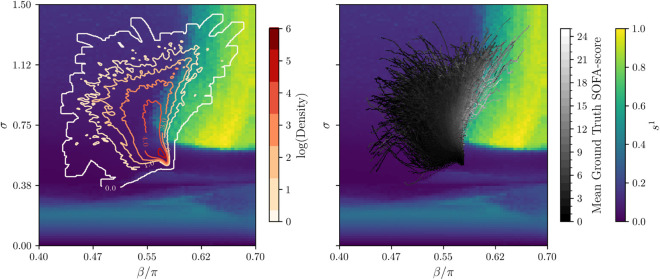
**(A)** Contour lines of predicted latent point distribution (β,σ) overlaid to the physical parameter space, cf. Figure 5A. The parameter space is colored with the values of the normalized desynchronization metric $s^{1}\left(\beta ,\sigma \right)$ , where brighter values indicate larger desynchronization. The lines are colored by density, with red values indicating greater density. **(B)** Shows the same parameter space, but the overlay points of the ground-truth SOFA score colored in grayscale; here, it is desired that the color gradients align.

Figure 10A shows contour lines of the predicted sample density of generated (β,σ) positions overlaid to the model parameter space, where both the smooth low-desynchronized area between β -values 0.4 and 0.5 and the more dynamic highly desynchronized area between β -values 0.55 and 1.0 are occupied. The lower-right region of the parameter space remains unused. The distribution strongly centers around the point $\left(\beta \approx 0.57,\sigma \approx 0.57\right)$ ; from there, individual trajectories spread out into all directions.

In Figure 10B, the predicted latent positions colored by the (mean) ground-truth SOFA scores are overlaid to the model parameter space. Brighter background colors indicate higher desynchronization values $s^{1}\left(\beta ,\sigma \right)$ , corresponding to more pathological organ states; similarly, higher ground-truth SOFA scores are indicated by brighter colors. Therefore, ideally, the color gradients match. The general systematics align, indicating that the Latent Dynamics Model systematically uses the parameter space of the physiological network model to express organ system state.

**FIGURE 11:**
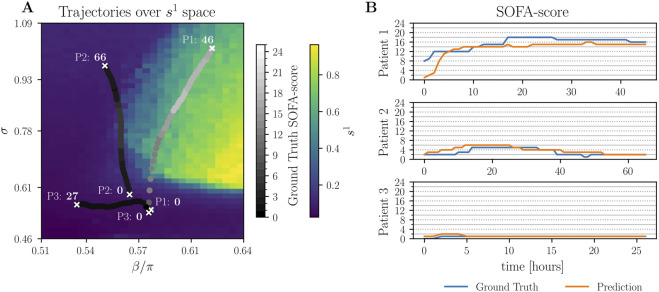
**(A)** Three patient trajectories in the physical parameter space (β,σ) , with ground-truth SOFA scores in grayscale, brighter values indicating worse measured organ functionality. Timestamps mark trajectory beginning and end. The background heatmap shows $s^{1}$ values (right colorbar), with brighter colors indicating higher desynchronization and worse predicted organ function. **(B)** Time series comparing predicted (orange) versus ground-truth (blue) SOFA scores for the same patients.

In Figure 11, three hand-picked examples illustrate possible physiological progressions and their representation inside the physical parameter space, with predicted and ground-truth SOFA score time evolution in Figure 11B, with the left panel Figure 11A showing the corresponding latent trajectories colored by the ground-truth values.

Patient 1 has, at intensive care unit admission t=0 , a relatively high SOFA score of 8, and the organ system deteriorates further, ultimately increasing to a value of 18 and subsequently lowering again. In the beginning, the prediction underestimates the severity of Patient 1’s condition by 7 score points, yet it picks up the trend of deterioration. This progression is mirrored in the parameter space, where the points clearly move with large steps from darker background colors (more synchronization, better organ functionality) to brighter colors (less synchronization, worse organ functionality).

Similarly, Patient 2 has an initial SOFA score of 2, illustrating a recovery trajectory, where the patient’s condition shortly worsens after admission but decreases gradually thereafter. The model tracks this directional change, although with imprecise magnitude estimates and shifted prediction timings, reflected in the latent trajectory which initially moves toward the brighter region, cuts it for a few time-steps and then enters back into the darker zone as recovery progresses.

Patient 3 arrives with a SOFA score of 0, exhibiting a slightly increased score the following days but remaining at a low value throughout their stay. Initially, the latent trajectory of Patient 3 shortly moves toward the boundary of the desynchronized region, followed by a turn away into the darker, synchronized region.

Overall, these three exemplary patients show that the model can capture diverse physiological trajectories within the physiological network model parameter space. Although the magnitude of predicted SOFA scores sometimes deviates from ground truth, the model consistently captures directional trends, such as deterioration, recovery, and saturation, through meaningful movement in the parameter space. Performance varies across patients, as reflected by the deviations in Figure 9 and heterogeneity of trajectories in Figure 10B. Importantly, interpretability operates at the trajectory level rather than the coordinate level: what matters is not the absolute position of a patient in the (β,σ) space, but the direction and magnitude of movement relative to the synchronization landscape, which serves as a physiologically grounded proxy for organ dysfunction.

## 6 Discussion

The quantitative results in Table 3 demonstrate that the proposed Latent Dynamics Model is a suitable method for sepsis risk assessment. Combined with the physiological network model, the method outperforms YAIB baselines in terms of AUROC (84.08%) and AUPRC (9.91%) , whereas the best baseline model, GRU, achieved 83.6% AUROC and 9.1% AUPRC. Notably, the Latent Dynamics Model differs from the baseline models in multiple ways beyond the physiological network model integration. The proposed architecture decomposes the prediction task according to the Sepsis-3 definition, with separate modules for infection and organ failure assessment, allowing to observe organ deterioration even when a patient is already diagnosed with sepsis. Additionally, the architecture includes an encoder for latent representation and a decoder to encourage meaningful latent space structure.

The physiological network model provides important inductive bias to the learned latent space. Unlike purely data-driven machine learning approaches, where interpretability of predictions is achieved exclusively through *post-hoc* methods, the interpretation of our latent space is established *a priori*. The trajectory-based representation of patients in the latent space aligns with clinician preferences identified in prior work by Eini-Porat et al. (2022), where survey participants emphasized that temporal trends in patient trajectories should be the prediction target and expressed preference for trajectory-based outputs over binary event predictions. Although the physiological network model parameters β (biological age including comorbidities) and σ (organ–immune interaction strength) do not correspond to directly observable physiological quantities, this study demonstrates how statistical learning can be combined with mechanistic modeling to enhance interpretability.

Numerical results suggest that the physiological network model is capable of describing sepsis dynamics. The descriptive ability of the physiological network model may be further improved by considering both network layers (namely, using not only $s^{1}$ but also $s^{2}$ ). Notably, only a limited portion of the complete (β,σ) parameter space was utilized for prediction, specifically the region containing a smooth monotonic transition. Regions of sharp changes were deliberately avoided, as shown in Figure 5.

**FIGURE 12:**
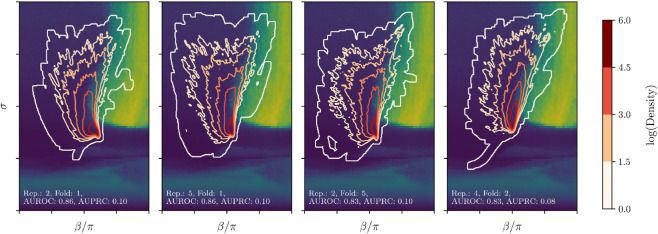
Density of latent points of the two best performing models (left two panels) and worst performing models (right two panels), based on the test AUROC, overlaid to the physiological network model parameter space. Red colors indicate higher concentration of points. For this plot, the background has been dimmed, to increase distribution visibility.

The mapping from patient observations to latent parameters is inherently non-unique: different training runs converge to distinct yet equally valid solutions. This ambiguity is a consequence of projecting high-dimensional electronic healthcare record data onto a two-dimensional physiological parameter space, where many embeddings can satisfy the learning objective equally well. The multi-objective nature deepens this further, likely introducing a complex optimization landscape without a single global optimum, but rather a Pareto frontier of trade-offs, making convergence to any particular solution sensitive to initialization. Figure 12 illustrates this concretely, showing how latent representations vary across cross-validation folds: although predictive performance remains comparable, the variability exceeds that of the baseline model (Table 3), and the distribution of patient trajectories in latent space shifts moderately. Learned representations should, therefore, not be treated as definitive but interpreted with awareness that any single trained model reflects one of many valid solutions.

Future research may address the following points:

- Attribution of the individual components in the proposed method to the resulting predictive performance. This includes systematic ablation studies of architectural components and exploration of alternative latent representations to assess robustness of the learned predictive structure. We further investigate whether modifications to the model design can enhance the predictive utility of the organ-dysfunction module.
- External validation and training on additional clinical datasets to assess generalizability, which is currently ongoing.
- Extension of the physiological network model with a temporal component or adapting the rollout strategy of the Latent Dynamics Model, thereby shifting to the offline prediction setting of sepsis assessment.
- Deeper analysis of the learned mapping, for example, whether the regularization term $d_{\theta }$ effectively structures the parameter space in clinically meaningful ways and whether individualized latent representations could support treatment decisions.
- Exploring the data split characteristics that drive the performance variability.

In conclusion, this study has motivated and demonstrated that hybrid modeling, combining physiologically grounded dynamics with data-driven learning, can address the limitations of purely mechanistic and purely black-box approaches to sepsis onset prediction.

Building on the physiological network model by Sawicki et al. (2022), Berner et al. (2022), which represents organ and immune interactions and has not previously been tested on clinical data, here we have demonstrated that it provides a viable latent structure for sepsis onset prediction. To this end, we introduce the Latent Dynamics Model, a neural architecture inspired by physics-informed machine learning that embeds this physiological framework into a deep-learning pipeline.

Evaluated on retrospective clinical data, the model achieves an AUROC of 84.08 % and AUPRC of 9.91% , delivering competitive performance while embedding a physiologically grounded latent structure. The inclusion of the physiological prior enables interpretation of patient trajectories in the latent space, revealing clinically plausible patterns of deterioration, defined as any increase in the SOFA score, and recovery, offering a richer clinical insight than a single opaque risk estimate.

Overall, this study establishes hybrid physics-informed deep learning as a promising pathway for interpretable clinical decision support, offering an alternative to purely black-box models while maintaining competitive predictive performance.

## Data Availability

The data used in this work, the MIMIC-IV database, is a publicly available, de-identified dataset. Access was granted following completion of the required training and data use agreement via PhysioNet. The code for cohort construction and computer simulations will be provided in a public repository upon publication https://github.com/unartig/sepsis osc; further inquiries can be directed to the corresponding author.

## Footnotes

1 Differences in patient numbers arise from underlying database versions (2.2 as opposed to 2.0 in YAIB).
