# Supplementary material for: Combining machine learning and physiological network models for sepsis prediction
Source: Front Netw Physiol. 2026 Jun 19;6:1852577. doi: 10.3389/fnetp.2026.1852577 (PMC13327882; doi:10.3389/fnetp.2026.1852577)
Supplement: Supplementary file 1 [file DataSheet1.pdf]

Supplementary Material

1 IMPLEMENTATION OF THE COMPUTER SIMULATION OF THE PHYSIOLOGICAL NETWORK MODEL

This Supplementary Material describes the implementation of the numerical integration of the physiological network model, and the choice of initial parameter values. The goal of this implementation is to reproduce the numerical results presented in Berner et al. (2022).

The backbone for the presented numerical integration is JAX Bradbury et al. (2018), a Python package for high-performance array computation, similar to NumPy or MATLAB but designed for automatic differentiation, vectorization and JIT. JIT-compilation and vectorization allow high-level numerical code to be translated to highly optimized accelerator-specific machine code, for example GPU. This way, performance benefits of massively parallel hardware can be utilized with minimal extra programming cost. For the actual integration a differential equation solver from diffrax Kidger (2022) was used, which provides multiple solving schemes fully built on top of JAX.

While Berner et al. (2022) uses a fourth-order Runge-Kutta method and a fixed step-size, this implementation[1] uses the Tsitouras 5/4 Runge-Kutta method Tsitouras (2011) with adaptive step-sizing controlled by a PID controller, allowing for more efficient integration while keeping similar accuracy. A relative tolerance of 10^{-3} and an absolute tolerance 10^{-6} were chosen. All simulations were carried out in 64-bit floating point precision, necessary for accurate and stable system integration.

Because of the element-wise differences used in the coupling terms $\varphi_i^{1,2} - \varphi_j^{1,2} \in \mathbb{R}^{N \times N}$ the computational cost scales quadratically with the number of oscillators N. These differences are then transformed by the computationally expensive trigonometric $\sin$ routine. To accelerate integration, these trigonometric evaluations were optimized following Böhle et al. (2022). Terms in the form $\sin(\theta_l - \theta_m)$ were expanded as:

$$\sin(\theta_l - \theta_m) = \sin(\theta_l)\cos(\theta_m) - \cos(\theta_l)\sin(\theta_m) \quad \forall l, m \in \{1, \dots, N\} \tag{S1}$$

By caching the terms $\sin(\theta_l)$, $\sin(\theta_m)$, $\cos(\theta_l)$, $\cos(\theta_m)$ once per iteration, the number of trigonometric evaluations per iteration is reduced from $2 \cdot [N\,(N-1)]$ to $2 \cdot [4N]$, significantly improving performance for mid to large oscillator populations.

In order to reproduce the computer simulations of Sawicki et al. (2022) and Berner et al. (2022), we choose the same parameter values: $\omega^1 = \omega^2 = \omega = 0$ (co-rotating frame). The phase lag parameters are $\alpha^{12} = \alpha^{21} = 0$, $\alpha^{11} = \alpha^{22} = -0.28\pi$. The slow adaptation rates are $\epsilon^1 = 0.03$ and $\epsilon^2 = 0.3$. The number of oscillators per layer is $N = 200$. As in Berner et al. (2022), the parameters β and σ are varied.

The initial conditions for κ_{ij}^1 and $\varphi_i^{1,2}/\pi$ are chosen randomly, see Fig. 3 of the main text. In order to model sepsis Berner et al. (2022), for the immune layer (κ_{ij}^2) an initial cytokine activation described by a two-frequency cluster state is chosen: a smaller cluster of CN oscillators and a bigger cluster of $(1-C)\,N$ elements, here $C = 0.2$. The coupling weights between the two clusters are zero. The size of the clusters C has no large impact. All initializations and parameter values can be found in Table S1.

[1] The code is available at `https://github.com/unartig/sepsis_osc/tree/main/src/sepsis_osc/dnm`

Symbol	Value	Symbol	Value
**Variables**			
φ_i^1	$\mathcal{U}\,[0, 2\pi)$	$\kappa_{i\neq j}^1$	$\mathcal{U}\,(-1, 1)$
φ_i^2	$\mathcal{U}\,[0, 2\pi)$	$\kappa_{i\neq j}^2$	clusters of size C and $1 - C$
**Parameters**			
M	50	N	200
C	20%	β	$[0.0, 1.0]\,\pi$
σ	$[0.0, 1.5]$	α^{11}, α^{22}	-0.28π
α^{12}, α^{21}	0.0	ω_1, ω_2	0.0
ϵ^1	0.03	ϵ^2	0.3

Table S1. Parameter values and initialization used for the numerical integration.

To average out the random initial values, M simulations are performed for each parameter set. Throughout this work an ensemble size of $M = 50$ was used.

2 SUPPLEMENTARY TABLES AND FIGURES

2.1 Figures

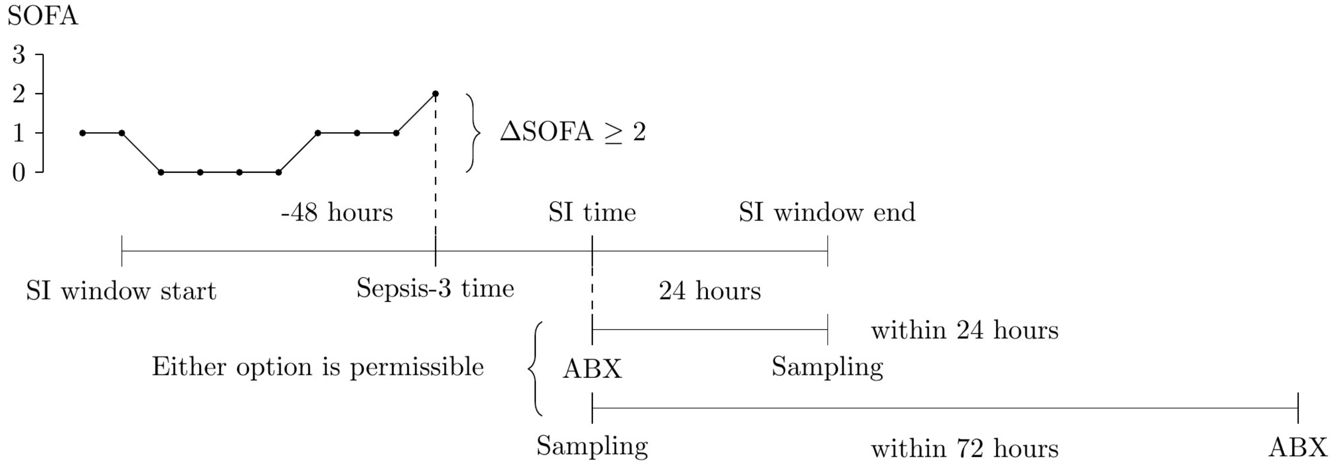

Figure S1. Graphical representation of the timings in the Sepsis-3 definition, taken from Bennett et al. (2025).

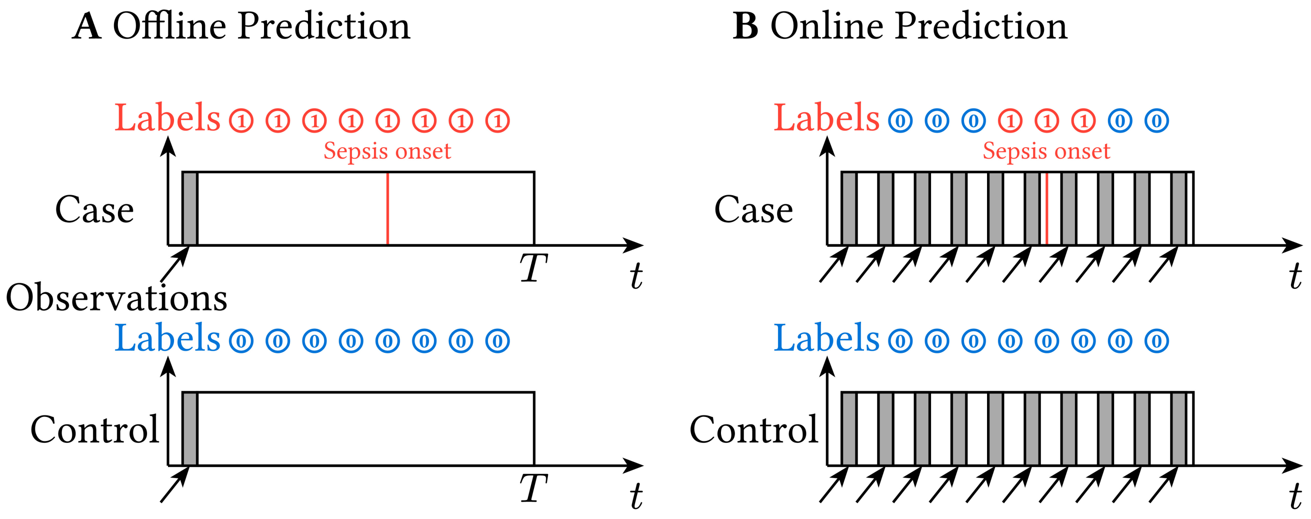

Figure S2. Illustration of the two predictions schemes, *offline* (**A**) vs. *online* (**B**) (figure heavily inspired by Moor et al. (2021)). The main difference is the sepsis labeling, as well as provision and utilization and arrival of observation data.

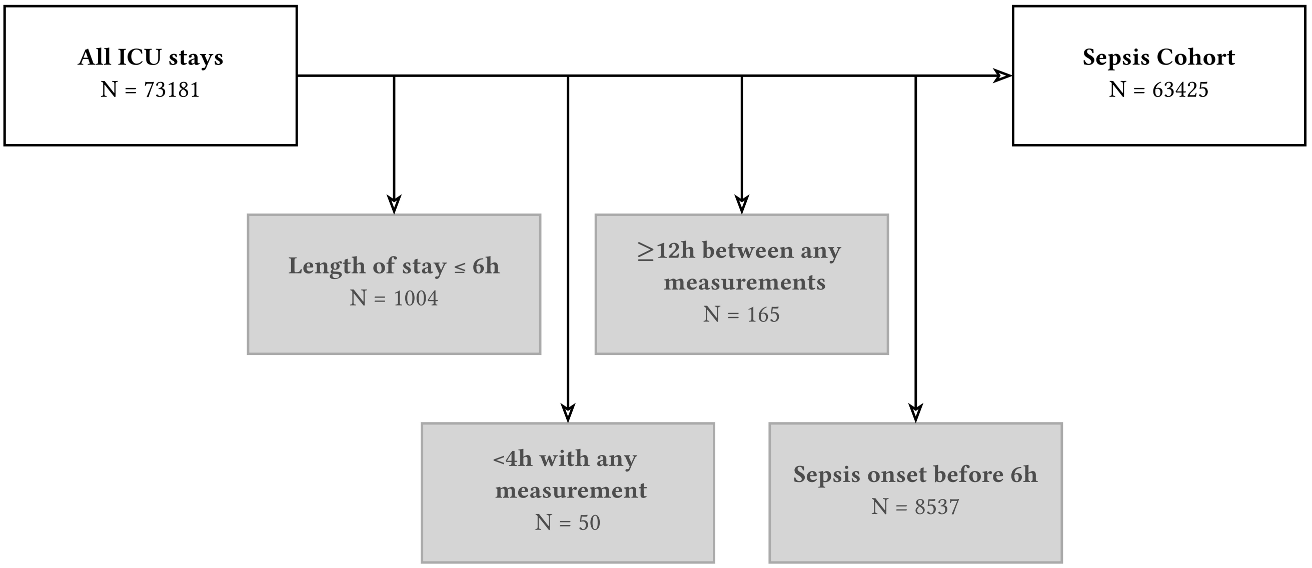

Figure S3. Cohort exclusion process.

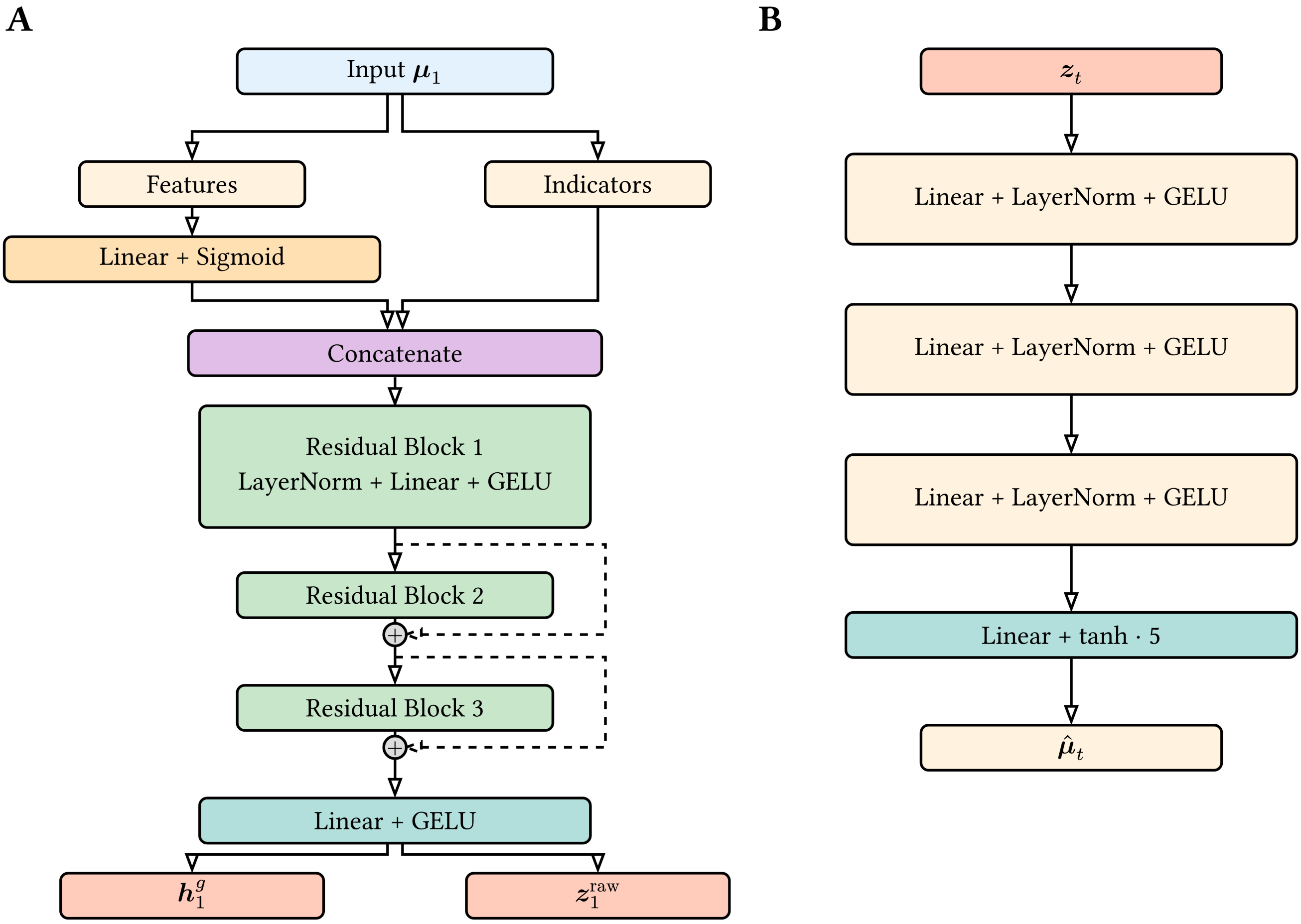

Figure S4. **A** Shows the initial latent encoder g_θ architecture with feature gating and residual connections (dashed arrows), with GELU activation Hendrycks and Gimpel (2023) and Layer-Normalization Ba et al. (2016). **B** Shows the decoder d_θ architecture, reconstructing only the features but not the imputation indicators.

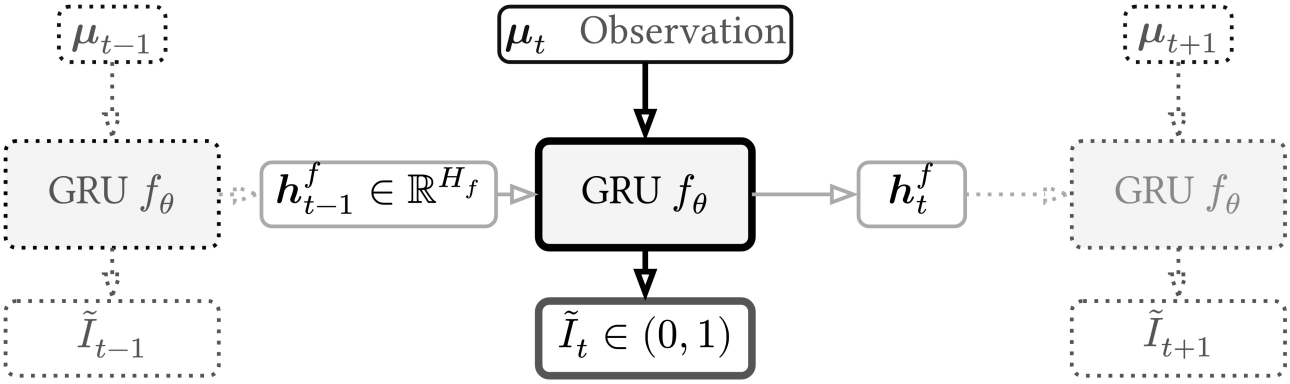

Figure S5. Schematic of the Infection Indicator Module architecture and rollout. The GRU-cell processes the electronic health record sequence $\mu_{1:t}$ step-by-step, propagating $\mathbf{h}_t$ to capture temporal dependencies, and outputs infection risk estimates $\widetilde{I}_t$ at each timestep. Here the GRU-block implicitly includes the down-projecting layer.

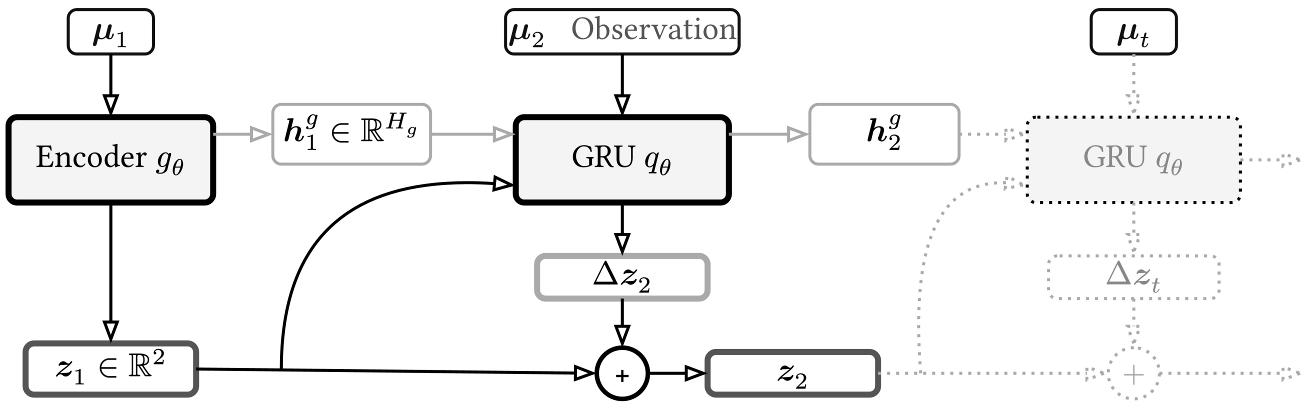

Figure S6. Schematic of the online-prediction rollout by the SOFA predictor module. The Encoder g_θ^e, generates the initial latent position $\hat{\mathbf{z}}_1^{\text{raw}}$ based on the first observed electronic health record $\boldsymbol{\mu}_1$. Afterwards, the GRU-cell processes the following electronic health record sequence $\boldsymbol{\mu}_{1:t}$ step-by-step, maintaining $\mathbf{h}_t$ to capture temporal dependencies, and outputs the change in latent position $\Delta\hat{\mathbf{z}}_t$ at each timestep. The new position is the sum of the previous position and its update $\hat{\mathbf{z}}_{t-1} + \Delta\hat{\mathbf{z}}_t$. Here the GRU-block implicitly includes the down-projecting layer. The down-projecting layer has been initialized via the QR-decomposition, to create unbiased random orthonormal weights, and scaled initially by a factor of 0.1.

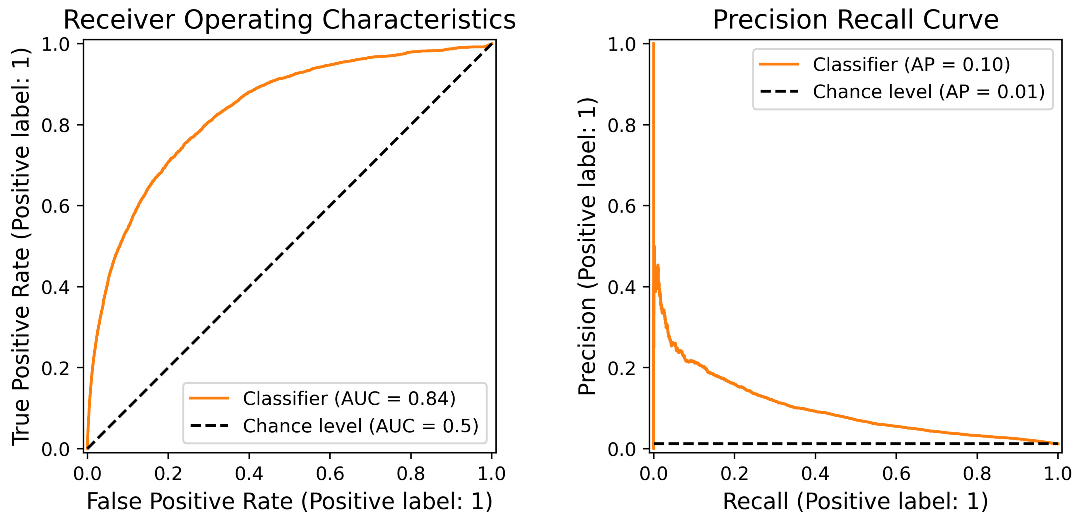

Figure S7. Receiver Operating Characteristic and precision-recall curves generated by sweeping the detection threshold.

2.2 Tables

ricu-Name	Unit	Min	Max	Description	% Missing
age	years	0	100	patient age	0.00
sex				patient sex	0.00
height	cm	10	230	patient height	48.70
weight	kg	1	500	patient weight	7.19

Table S2. Static input features for the prediction task, with missingness information per stay.

ricu-Name	Unit	Min	Max	Description	% Missing
alb	g/dL	0	6	albumin	99.16
alp	['IU/L', 'U/l']	0		alkaline phosphatase	98.46
alt	['IU/L', 'U/l']	0		alanine aminotransferase	98.44
ast	['IU/L', 'U/l']	0		aspartate aminotransferase	98.42
be	['mEq/L', 'mmol/l']	-25	25	base excess	95.20
bicar	['mEq/L', 'mmol/l']	5	50	bicarbonate	93.28
bili	mg/dL	0	100	total bilirubin	98.43
bili_dir	mg/dL	0	50	bilirubin direct	99.87
bnd	%			band form neutrophils	99.79
bun	mg/dL	0	200	blood urea nitrogen	93.23
ca	mg/dL	4	20	calcium	94.03
cai	mmol/L	0.50	2	calcium ionized	96.93
ck	['IU/L', 'U/l']	0		creatine kinase	99.08
ckmb	ng/mL	0		creatine kinase MB	99.07
cl	['mEq/L', 'mmol/l']	80	130	chloride	92.90
crea	mg/dL	0	15	creatinine	93.21
crp	mg/L	0		C-reactive protein	99.95
dbp	['mmHg', 'mm Hg']	0	200	diastolic blood pressure	13.33
fgn	mg/dL	0	1500	fibrinogen	99.27
fio2	%	21	100	fraction of inspired oxygen	91.32
glu	mg/dL	0	1000	glucose	91.47
hgb	g/dL	4	18	hemoglobin	93.53
hr	['bpm', '/min']	0	300	heart rate	7.29
inr_pt				prothrombin time/international normalized ratio	95.59
k	['mEq/L', 'mmol/l']	0	10	potassium	92.73
lact	mmol/L	0	50	lactate	96.81
lymph	%	0	100	lymphocytes	99.15
map	['mmHg', 'mm Hg']	0	250	mean arterial pressure	11.98
mch	pg	0		mean cell hemoglobin	93.67
mchc	%	20	50	mean corpuscular hemoglobin concentration	93.67
mcv	fL	50	150	mean corpuscular volume	93.67
methb	%	0	100	methemoglobin	99.99
mg	mg/dL	0.50	5	magnesium	93.52
na	['mEq/L', 'mmol/l']	110	165	sodium	92.83
neut	%	0	100	neutrophils	99.15
o2sat	['%', '% Sat.']	50	100	oxygen saturation	9.36
pco2	['mmHg', 'mm Hg']	10	150	CO2 partial pressure	95.20
ph		6.80	8	pH of blood	94.51
phos	mg/dL	0	40	phosphate	93.99
plt	['K/uL', 'G/l']	5	1200	platelet count	93.55
po2	['mmHg', 'mm Hg']	40	600	O2 partial pressure	95.51
ptt	sec	0		partial thromboplastin time	95.30
resp	['insp/min', '/min']	0	120	respiratory rate	8.27
sbp	['mmHg', 'mm Hg']	0	300	systolic blood pressure	13.31
temp	['C', '°C']	32	42	temperature	73.12
tnt	ng/mL	0		troponin t	99.23
urine	mL	0	2000	urine output	48.45
wbc	['K/uL', 'G/l']	0		white blood cell count	93.66

Table S3. Dynamic input features for the prediction task, with missingness information per hour. As most of the lab measurements are rarely updated, the high missingness values occur.

Characteristic	All patients	SEP-3 positive	SEP-3 negative
**Demographics**			
N	63425 (100.0)	3320 (5.2)	60105 (94.8)
Male	35170 (55.5)	1881 (56.7)	33289 (55.4)
Age at admission	65.0 (53.0–76.0)	65.0 (54.0–76.0)	65.0 (53.0–76.0)
Weight at admission	77.6 (65.1–92.3)	77.6 (65.6–94.0)	77.6 (65.0–92.2)
**Clinical Outcomes**			
SOFA median	3.0 (1.0–5.0)	3.0 (1.0–5.0)	3.0 (1.0–5.0)
SOFA max	4.0 (2.0–6.0)	5.0 (4.0–8.0)	4.0 (2.0–6.0)
Hospital LOS hours	157.7 (92.8–268.9)	335.1 (194.2–548.6)	150.3 (90.9–256.0)
Hospital Mortality	4828 (7.6)	879 (26.5)	3949 (6.6)
Sepsis-3 onset time	-	13.0 (8.0–34.0)	-
**Ethnicity**			
White	40364 (63.6)	2087 (62.9)	38277 (63.7)
Black	5809 (9.2)	262 (7.9)	5547 (9.2)
Asian	721 (1.1)	42 (1.3)	679 (1.1)
Hispanic	630 (1.0)	32 (1.0)	598 (1.0)
Other/Unknown	14924 (23.5)	897 (27.0)	14027 (23.3)
**Admission Type**			
Medical	45009 (71.0)	2817 (84.8)	42192 (70.2)
Surgical	2239 (3.5)	45 (1.4)	2194 (3.7)
Other/Unknown	15200 (24.0)	458 (13.8)	14742 (24.5)

Table S4. Characteristics and demographics of the cohort. Numerical variables are summarized by *median [Interquantile Range 25th - 75th percentile]* and numerical variables by incidence (%)

Name	Shape	Count
**General**		
T_D	(1,)	1
tau_causal_decay	(1,)	1
decision_thresh_d	(1,)	1
decision_thresh_m	(1,)	1
	**Total:**	**4**
**Infection Module**		
h_init	(64,)	64
GRU weight_ih	(192, 104)	19,968
GRU weight_hh	(192, 64)	12,288
GRU bias	(192,)	192
GRU bias_n	(64,)	64
linear_proj_out weight	(1, 64)	64
linear_proj_out bias	(1,)	1
	**Total:**	**32,641**
**Decoder Module**		
linear0 weight	(16, 2)	32
linear0 bias	(16,)	16
linear1 weight	(16,)	16
linear1 bias	(16,)	16
linear3 weight	(32, 16)	512
linear3 bias	(32,)	32
linear4 weight	(32,)	32
linear4 bias	(32,)	32
linear6 weight	(32, 32)	1,024
linear6 bias	(32,)	32
linear7 weight	(32,)	32
linear7 bias	(32,)	32
linear9 weight	(52, 32)	1,664
linear9 bias	(52,)	52
	**Total:**	**3,524**

Name	Shape	Count
**SOFA Module (Encoder)**		
gating weight	(52, 52)	2,704
norm1 weight	(104,)	104
norm1 bias	(104,)	104
linear1 weight	(64, 104)	6,656
linear1 bias	(64,)	64
norm2 weight	(64,)	64
norm2 bias	(64,)	64
linear2 weight	(64, 64)	4,096
linear2 bias	(64,)	64
norm3 weight	(64,)	64
norm3 bias	(64,)	64
linear3 weight	(64, 64)	4,096
linear3 bias	(64,)	64
linear4 weight	(16, 64)	1,024
linear4 bias	(16,)	16
linear_z weight	(2, 16)	32
linear_z bias	(2,)	2
linear_h weight	(8, 16)	128
linear_h bias	(8,)	8
	**Total:**	**19,418**
**SOFA Module (RNN)**		
GRU weight_ih	(24, 106)	2,544
GRU weight_hh	(24, 8)	192
GRU bias	(24,)	24
GRU bias_n	(8,)	8
linear_proj_out weight	(2, 8)	16
	**Total:**	**2,784**

Table S5. Detailed parameter count of the latent dynamics model modules.

REFERENCES

Ba, J. L., Kiros, J. R., and Hinton, G. E. (2016). Layer normalization. *arXiv preprint arXiv:1607.06450*

Bennett, N., Plečko, D., and Ukor, I.-F. (2025). Sepsis 3 label — sep3. *Github repository* Accessed: 2025-10-07

Berner, R., Sawicki, J., Thiele, M., Löser, T., and Schöll, E. (2022). Critical parameters in dynamic network modeling of sepsis. *Frontiers in Network Physiology* 2. doi:10.3389/fnetp.2022.904480

Bradbury, J., Frostig, R., Hawkins, P., Johnson, M. J., Leary, C., Maclaurin, D., et al. (2018). JAX : composable transformations of python + numpy programs. *Github repository*

Böhle, T., Kuehn, C., and Thalhammer, M. (2022). On the reliable and efficient numerical integration of the kuramoto model and related dynamical systems on graphs. *International Journal of Computer Mathematics* 99, 31–57. doi:10.1080/00207160.2021.1952997

Hendrycks, D. and Gimpel, K. (2023). Gaussian error linear units (gelus). *arXiv preprint arXiv:1606.08415*

Kidger, P. (2022). On neural differential equations. *arXiv preprint arXiv:2202.02435*

Moor, M., Rieck, B., Horn, M., Jutzeler, C. R., and Borgwardt, K. (2021). Early prediction of sepsis in the ICU using machine learning: A systematic review. *Frontiers in Medicine* Volume 8 - 2021. doi:10.3389/fmed.2021.607952

Sawicki, J., Berner, R., Löser, T., and Schöll, E. (2022). Modeling tumor disease and sepsis by networks of adaptively coupled phase oscillators. *Frontiers in Network Physiology* 1. doi:10.3389/fnetp.2021.730385

Tsitouras, C. (2011). Runge–kutta pairs of order 5 (4) satisfying only the first column simplifying assumption. *Computers & Mathematics with Applications* 62, 770–775
